# In-depth characterization of the Ca_V_2.2-knockout mouse line

**DOI:** 10.1038/s41598-026-60827-w

**Published:** 2026-07-09

**Authors:** Katharina Wintz, Felix Schumacher, Clemens Pfeiffer, Maja Schäfer, Ian Gering, Sarah Schemmert, Antje Willuweit, Janine Kutzsche

**Affiliations:** 1 Institut für Physikalische Biologie, Faculty of Mathematics and Natural Sciences, Heinrich Heine University Düsseldorf, Universitätsstraße 1, 40225 Düsseldorf, Germany; 2 Institute of Biological Information Processing, Structural Biochemistry (IBI-7), Forschungszentrum Jülich GmbH, Wilhelm-Johnen-Straße, 52428 Jülich, Germany; 3 Institute of Neuroscience and Medicine, Medical Imaging Physics (INM-4), Forschungszentrum Jülich GmbH, Wilhelm-Johnen-Straße, 52428 Jülich, Germany

**Keywords:** Characterization study, Ca_V_2.2 calcium channel, Knockout, Behavior, Seizures, Neurology, Neuroscience

## Abstract

The voltage-gated calcium channel Ca_V_2.2 is predominantly located in neuronal synapses, where it plays a role in pain signaling and neurotransmitter release. Furthermore, Ca_V_2.2 is important during early central nervous system development, as it is subsequently replaced by other calcium channels. A number of mouse lines with disrupted Ca_V_2.2 channels have been developed through the utilization of a gene-targeting vector. The Ca_V_2.2-deficient mice primarily exhibited reduced anxiety-linked behavior and response to pain stimuli. In a previous study, we observed hyperactivity and seizures in Ca_V_2.2-knockout mice. Given the absence of prior descriptions of these characteristics, an in-depth characterization was conducted. This characterization included longitudinal behavioral studies, as well as histological, proteomic, and metabolomic analyses. Irrespective of the frequency with which testing was conducted, a variety of behaviors were observed (e.g., hyperactivity and increased exploratory behavior) in comparison with wild-type mice. The frequency and severity of the performed tests demonstrated a positive correlation with the magnitude of the difference in molecular markers between Ca_V_2.2-knockout and wild-type mice. Seizures were observed but were not common. These results suggest that the Ca_V_2.2-knockout mouse lines are susceptible to repeated stress stimuli during experimental studies. The mice are characterized by hyperactivity, explorative behavior, and sometimes experience seizures.

**Supplementary Information:**

The online version contains supplementary material available at 10.1038/s41598-026-60827-w.

## Introduction

The family of voltage-gated calcium channels (VGCCs) consists of multiple ion channels that vary in tissue localization and voltage sensitivity. These channels can be categorized into two classes: high-voltage-activated channels (Ca_V_1.x L-type, Ca_V_2.1 P/Q-type, Ca_V_2.2 N-type, and Ca_V_2.3 R-type) and low-voltage-activated channels (Ca_V_3.x T-type). All VGCCs are activated by membrane voltage changes, which allow calcium ions (Ca^2+^) to enter the cell and trigger signaling pathways. These channels are comprised of an α_1_-subunit and auxiliary α_2_δ-, β-, and occasionally γ-subunits. The α_1_-subunit has been demonstrated to be responsible for pore-forming and specificity of the channel subtype^1^.

The neuronal Ca_V_2.2 N-type channel, which has an α_1B_-subunit encoded by the *CACNA1B* gene, is found in the dendrites and presynaptic terminals of neurons. Upon reaching the presynapse, the action potential triggers the influx of Ca^2+^ and subsequently the fusion of neurotransmitter-carrying vesicles with the presynaptic membrane. The Ca_V_2.2 channels have been identified as playing a role in the transmission of neuropathic pain and anxiety-related behaviors^2^. Moreover, the channel has been identified in microglia and astrocytes, where it potentially modulates Ca^2+^ signaling in inflammation^3,4^.

An R1389H (rs184841813) missense mutation in the *CACNA1B* gene has been associated with a myoclonus-dystonia syndrome, accompanied by cardiac arrhythmia that is triggered by generating smaller single-channel N-type currents^5^. Another group reported the presence of several biallelic loss-of-function mutations in children that resulted in epileptic encephalopathies and hyperkinetic movement disorders^6^. The *CACNA1B* gene has also been linked to a variety of psychiatric conditions including schizophrenia, autism spectrum disorders, and major depressive disorders^7^.

The Ca_V_2.2 channel is blocked specifically by the peptide antagonist ω-conotoxin MVIIA, which has been extracted from the venom of marine cone snails (genus *Conus*). The analgesic drug ziconotide is based on this peptide and is used to treat chronic pain^8^. Treatment with MVIIA is 100 times more effective than morphine, is not addictive, and is associated with serious adverse effects, including dizziness, nystagmus, somnolence, abnormal gait, and ataxia^9^. In vivo administration of MVIIA led to hypotension and tachycardia in rabbits; two of them also exhibited mild to severe tremors and bradycardia^10^. Intracerebroventricular injection in rats resulted in degeneration of neurons in the CA3 region of the hippocampus^11^.

Two Ca_V_2.2 knockout (KO) mouse lines are most commonly utilized in animal studies^12,13^. The line developed by Saegusa and colleagues exhibited reduced anxiety-related behaviors, as evidenced by a greater number of entries into the open arms in the zero maze, hyperactivity leading to greater distances moved in the open field test (OFT), and a reduced acoustic startle response. Additionally, a diminished reaction to inflammatory and neuropathic pain was observed. The group reported a lethality of up to 30% in homozygous offspring by the time the mice were weaned^13^. Ino and colleagues demonstrated reduced sympathetic nerve activity, elevated arterial blood pressure, and an accelerated heart rate in an alternative Ca_V_2.2 KO line^12^, thereby implicating the Ca_V_2.2 channel in the sympathetic regulation of the cardiovascular system^14^. These mice exhibited a form of hyperactivity, as measured by increased movement during the dark phase of the OFT, a stronger response to novel stimuli, and differences in vigilance state^15^. A subsequent neurochemical analysis revealed decreased levels of dopamine and 5-hydroxytryptamine (serotonin) in the striatum and frontal cortex^16^. In 2020, Saegusa and colleagues demonstrated that the KO of Ca_V_2.2 exclusively in microglia of a Parkinson’s disease mouse model led to reduced neuroinflammation during disease progression^17^. Similarly, we demonstrated a delayed disease onset and reduced inflammation when crossing a SOD1 mouse model (SOD1*G93A on a C57BL/6 J background) of amyotrophic lateral sclerosis with the Ca_V_2.2 KO mouse line by Saegusa et al. During the course of this study, we observed seizures in Ca_V_2.2 KO mice that had not yet been described for this line^18^.

Based on this observation, a more thorough examination of the Ca_V_2.2 KO mouse line was conducted. We performed behavioral tests at varying frequencies over time to determine whether elevated levels of experimental stress could induce seizures. Furthermore, we conducted histochemistry on brain tissue to assess the extent of potential neurodegeneration and inflammation. In order to investigate the consequences of Ca_V_2.2 KO on a molecular level, metabolomic and proteomic analyses were performed using mass spectrometry.

## Material and methods

### Mouse study

#### Mice

Heterozygous mice of the B6;129S4-Cacna1b^tm1Ttan^ line (RRID:IMSR_RBRC04884) were obtained from RIKEN (strain: RBRC04884, Riken BRC Experimental Animal Division). These mice were bred in-house with C57BL/6 J mice (RRID:IMSR_JAX:000,664) obtained from CRIVER (strain: 000,664, Charles River Laboratories). Genotyping of the mice was conducted using DNA extracted from ear tissue collected at P21. The Cacna1b genotype was determined using the RIKEN PCR protocol.

The mice were housed in the animal facility at the Forschungszentrum Jülich under controlled specific-pathogen-free conditions, including a 12/12-h light/dark cycle, humidity maintained at approximately 50%, and room temperature between 20 °C and 23 °C. The food pellets (Altromin) and tap water were autoclaved and available ad libitum. A maximum of five mice were housed in individually ventilated cages with standardized rodent bedding (Rettenmaier).

### Study design

The study included a total of 31 homozygous Ca_V_2.2 KO (Cav^−/−^) and 32 wild-type (WT) mice. These mice were obtained from other breeding strategies as surplus, which required that the study be conducted in two parts. Each genotype was divided further. The first cohort of 16 mice was utilized exclusively at the start (week 7) and end (week 19) of the study (15 mice for the Cav^−/−^ mice). The second cohort of 16 mice was utilized every two weeks to determine whether higher testing frequencies trigger seizures (high frequency = HF vs. low frequency = LF). The distribution of sex within each group and between the groups was equal (Cav^−/−^_LF 8♂:7♀, Cav^−/−^_HF 8♂:8♀, WT_LF 8♂:8♀, WT_HF 8♂:8♀). The study included the OFT, the object exploration test, the marble burying test, the elevated zero maze, and an additional nesting test at the start and end of the study period. Before each test, the mice were habituated in the experimentation room for 30 min in individual cages. The mice were weighed and examined for general health criteria on three days per week. Subsequently, the mice were euthanized to obtain blood and brain tissue. Anesthesia was induced using a ketamine and medetomidine mixture (100 mg/kg and 0.3 mg/kg, respectively). Terminal blood collection was performed via cardiac puncture, after which the mice were confirmed dead.

### Plasma and tissue collection

Subsequent to euthanization, the brains were dissected and cut into the two hemispheres. The left hemisphere was snap frozen in isopentane and stored at –‍80 °C until liquid chromatography-tandem mass spectrometry (LC–MS/MS) analysis. The right hemisphere was placed in 4% paraformaldehyde (PFA) for one to three days. Then, it was incubated in 30% sucrose for one day, and stored at –‍80 °C until histochemistry. Additionally, the blood was extracted from each mouse by cardiac puncture. The plasma was collected following a centrifugation at 3,000 xg for 15 min, and stored at –‍80 °C until LC–MS/MS analysis.

### Behavioral tests

#### Open field test

The mice were videotaped for 30 min in a cubical arena (45 cm x 45 cm). To assess their anxiety and exploratory behavior, the arena was divided into a center (22 cm x 22 cm) and a border zone. The videos were analyzed using EthoVision 15.0 software (Noldus, RRID:SCR_000441). Parameters such as exploration time, time spent moving, and total distance moved were determined. The OFT was repeated every two weeks.

#### Object exploration test

The same setup and analysis used for the OFT were also used for the object exploration test. Subsequent to the OFT, the mice were briefly removed from the arena, a cuboid object (6 cm x 6 cm x 15 cm) was placed in the center, and the mice were returned. The exploratory behavior was recorded for 10 min. The test was repeated every two weeks.

#### Elevated zero maze

The mice were placed on an elevated, circular platform with open and closed arms (diameter 60 cm, width 5 cm, height 70 cm). Their behavior was videotaped for 5 min. The videos were analyzed using EthoVision 15.0. The groups were compared based on the parameters of distance moved, time spent moving, and frequency of entering the open arms.

#### Marble burying test

Another test used to assess anxiety and obsessive–compulsive behavior is the marble burying test^19^. Ten marbles were placed in a fresh cage, which contained clean bedding. The mice were placed in the cage for 30 min. The marbles buried by at least ¾ were counted.

#### Nest building test

The mice were placed individually in a fresh cage containing an intact nestlet and remained there overnight. On the following day, they were returned to their respective littermates. The weight of the remaining nestlet and the quality score, ranging from zero (nestlet not touched) to five (perfect nest), were documented^20^. This test was only conducted at the start and the end of the study.

#### Statistics

First, differences between part 1 and part 2 of the study were analyzed by three-way ANOVA (factors: group x week x part). If a significant three-way interaction was detected, subsequent simple main effect tests were performed, followed by post-hoc Holm-Sidak tests. Significant results are displayed in the corresponding tables. If no significant three-way interaction was detected, a subsequent two-way ANOVA (factors: part × group, and part × week) was performed. When significant differences were detected, post-hoc Holm-Sidak tests were conducted to determine the influence of the different parts on group or week, which is displayed in the corresponding tables. The same procedure was applied to evaluate potential sex-dependent differences. This manuscript does not include details for the three-way and two-way ANOVAs performed for the influence of factors part and sex.

All statistical analyses for the behavioral tests were performed using Sigma Plot v.11.0 (Systat Software, RRID:SCR_003210). The software automatically tests normality prior to executing each parametric test. If normality was not observed, the software switched to non-parametric tests. The behavioral tests and body weight were analyzed using two-way repeated measures (RM) ANOVA, followed by post-hoc Holm-Sidak testing. The data were visualized using GraphPad PRISM v.5.00 (GraphPad Software, Inc., RRID:SCR_002798).

### Immunohistochemistry and western blots

#### Immunohistochemistry

The right brain hemispheres from six mice per group were sectioned into 20 μm-thick slices using a cryostat (CM3050 S Cryostat, Leica, RRID:SCR_020214). The slices were mounted on microscope slides and stored at –‍80 °C until the immunohistochemical analysis.

For brain histochemistry, the following markers were used on four slices per mouse: (a) neuronal nuclei (NeuN) and glial fibrillary acidic protein (GFAP); (b) Ionized calcium-binding adaptor molecule 1 (Iba1). An antigen retrieval was performed using 70% formic acid for 5 min for the fluorescent staining (a). The tissue was subsequently incubated overnight at 4 °C with the following primary antibodies: rabbit-anti-GFAP 1:1000 (Z0334, Agilent Dako, RRID:AB_10013382), mouse-anti-NeuN 1:1000 (MAB377, KGaA, RRID:AB_2298772). On the next day, the secondary antibodies (goat-anti-rabbit IgG (H + L), Alexa Fluor 488, A-11008, Invitrogen; goat-anti-mouse IgG (H + L), Alexa Fluor 568, A-11004, Invitrogen) were incubated for two hours at room temperature, and the nuclei were counterstained with DAPI. Between each step, the slides were washed with Tris-buffered saline containing 1% Triton X-100 (TBS-T) three times for 5 min each. For the fluorescent staining (b), antigen retrieval consisted of a 30 min incubation with 10 mM citrate buffer (pH 6) at 80 °C. The primary antibody rabbit-anti-Iba1 (019–19,741, FUJIFILM Wako, RRID:AB_839504) and the secondary antibody goat-anti-rabbit (Alexa Fluor 488, A-11008, Invitrogen) were used. The wash buffer for (b) was phosphate-buffered saline with 0.25% Triton X-100 (PBS-T).

#### Microscopy

Images of the cortex, hippocampus, thalamus, hypothalamus, and the medulla were obtained using the Leica LMD6000 microscope with the Leica Application Suite 4.0 software and the 10 × objective (Leica Mikrosysteme Vertrieb GmbH, Germany). For each staining, the images were taken on the same day and under the same conditions (GFAP: exposure time 360 ms, gain 1x, gamma 1.00; NeuN: exposure time 800 ms, gain 14x, gamma 1.20; Iba: exposure time 700 ms, gain 2x, gamma 1.30).

#### Quantification and statistics

The analyses of histochemistry results were conducted using ImageJ (version 1.50b^21^,, RRID:SCR_003070). NeuN counts were analyzed using a consistent region of interest (ROI) and the *Analyze Particles* function. The stained area by GFAP and Iba1 was analyzed using a consistent ROI and the *Analyze Area Fraction* function.

The histological results were analyzed using one-way ANOVA, followed by post-hoc Holm-Sidak testing. Results were considered significant at *p* < 0.05. The data were visualized using GraphPad PRISM v.5.00 (GraphPad Software, Inc., RRID:SCR_002798).

#### Western blots

The left brain hemispheres were homogenized in 1 mL of 20 mM Tris/250 mM NaCl buffer (pH 8), containing one Compleate EDTA free Protease Inhibitor Cocktail tablet (Roche) per 50 mL, using a Precellys Evolution homogenizer (Bertin Technologies) at 6000 rpm for 3 × 20 s, with a 5 s pause between each cycle. The samples were continuously cooled. The homogenates were stored at –‍80 °C until further use.

The homogenates were thawed on ice and mixed with double-distilled water (1:10). Lämmli buffer was added, and the samples were heated for 5 min at 95 °C. The 15% polyacrylamide/6% polyacrylamide gel containing the samples was run at 400 V and 45 mA for 45 min. The polyvinylidene fluoride membrane was activated in methanol, followed by a gel blot for 30 min in Bjerrum Schafer Nielsen buffer (48 mM Tris, 39 mM glycine, 20% methanol, pH 9.2). Ca_V_2.2 was detected using a rabbit-anti-CACNA1B primary antibody (ACC-002, Alomone Labs, RRID:AB_2039766) over night at 4 °C. After washing the blot with TBS/0.1% Tween three-times, the secondary antibody goat-anti-rabbit conjugated with horseradish peroxidase (31,460, Thermo Fisher Scientific, RRID:AB_228341) was incubated for one hour at room temperature. The blot was washed again and the channel was visualized using the ELC Select Western Blotting Detection Reagent kit (RPN2235, Cytiva).

### LC–MS/MS proteomic analysis

#### Sample preparation

The brain homogenates created under the section *Western blots* were used for the proteomic analysis. The bicinchoninic acid (BCA) assay kit (Thermo Fisher Scientific) was used to determine the total protein concentration. The brain homogenates were diluted 1:100 with PBS. 25 µL of the dilution were mixed with 200 µL of the kit’s working reagent in a 96-well plate, and the plate was then incubated for 30 min at 37 °C. The total protein concentrations were calculated from the acquired absorbance readings at 562 nm using a bovine serum albumin dilution series and a ClarioStar plate reader (BMG Labtech).

12 µg of protein from each hemisphere were mixed with 1% SDS/10 mM dithiothreitol (DTT), and incubated for 15 min at 90 °C. The samples were cooled to room temperature, mixed with 50 mM chloracetamide, and then incubated for 30 min in the dark. The alkylation reaction was quenched for 20 min using of 50 mM DTT. SP3 beads were added at a ratio of 1:20 (beads to total protein). The binding of proteins was achieved by the addition of ethanol to a final concentration of 80%. Subsequently, the beads were immobilized on a magnet, and the supernatant was discarded. The beads were rinsed twice with 400 µL 90% acetonitrile, and resuspended in 30 µL 100 mM HEPES/2.5 mM CaCl_2_ (pH 7.5). Trypsin Platinum (Promega) was added at a 1:100 ratio (enzyme to total protein), and the samples were incubated overnight at 37 °C. Following the enzymatic digestion, peptide cleanup via mixed-mode strong cation-reversed phase chromatography was performed using STAGE-Tips (Stop and Go Extraction) with sulfonated polystyrene-divinylbenzene Empore membranes (Sigma). STAGE-Tips were activated using 20 µL methanol, washed with 20 µL 0.1% formic acid/80% acetonitrile (buffer B), and washed twice with 20 µL 0.1% formic acid/water (buffer A). The samples were acidified to a final concentration of 2% using formic acid, and subsequently added to the STAGE-Tips. The tips were washed once with 30 µL buffer A, and twice with 30 µL buffer B. Tryptic peptides were eluted twice with 30 µL 5% ammonium hydroxide/60% acetonitrile and collected in low-binding tubes. The solvent was evaporated using a speed vac at 40 °C and 0.1 mbar (Christ). The peptides were reconstituted in 10 µL 10% acetonitrile.

#### Non-targeted proteomics

Proteomic analyses were conducted using the UltiMate 3000 RSCL nano-HPLC system (Thermo Fisher Scientific), which was coupled to an Impact II Q-TOF mass spectrometer (Bruker) with an acetonitrile-saturated nitrogen gas CaptiveSpray ion source (Bruker). 3 µL of freshly dissolved samples were loaded onto a µPAC reverse phase (RP) trap column (ThermoFisher Scientific) and separated using a 50 cm µPAC RP analytical column. Peptides were eluted at a flow rate of 600 nL/min using a gradient of buffer B (2%–‍30%). The effective separation time was 90 min, and the total run time per sample was two hours. The column temperature was maintained at 40 °C. Mass spectrometry data were acquired using the HyStar software (v.5.1, Bruker) in line-mode within a mass range of 200 m/z–‍1750 m/z. The acquisition rate was set to 5 Hz. The 14 most intense ions were selected for fragmentation, and fragment spectra were acquired automatically between 5 Hz–‍20 Hz, depending on the precursor intensity. Already-selected precursor ions were excluded for the next 0.4 min unless the signal-to-noise ratio improved threefold.

#### Data processing

The raw data files for each sample were converted to mzml files using MSConvert (v.3.0, Proteo Wizard^22^). Database searching and label-free quantification (LFQ) were performed using FragPipe (v. 22.0^23,24^,, RRID:SCR_022864). The LFQ-Match between runs workflow was selected and the Uniprot UP 000,000,589 *Mus musculus* (mouse) proteome was used as database. The MSFragger and IonQuant settings were left at their default values for tryptic peptides with alkylated cysteines.

#### Statistical analysis and visualization

Statistical analysis was performed using the online FragPipe-Analyst tool (v.1.13). The uploaded data were filtered for contaminant proteins and converted to a log_2_ scale. The samples were grouped by their genetic background, and missing values were imputed using the min method. A cutoff of an adjusted *p*-value of ≤ 0.05 along with a log_2_(FC) = 0 was applied to determine differentially expressed proteins. Volcano plots were generated to compare WT and Cav^−/−^ mice.

### LC–MS/MS metabolomic analysis

#### Sample preparation

The brain homogenates created under the section *Western blots* were used for the metabolomic analysis. The homogenates were stored at –‍20 °C for one hour and then centrifuged (15 min, 16,000 xg, 4 °C). The supernatant was evaporated using a vacuum concentrator 5301 (Eppendorf). The pellet was reconstituted in 100 μL water/methanol (1:1) and sonicated in an ice bath for one minute. Subsequent to a centrifugation at 4 °C and 16,000 xg for 15 min, the samples were ready for analysis.

Plasma samples were thawed on ice and briefly homogenized. 20 μL of plasma were mixed with 80 μL of ice-cold methanol. The mixture was vortexed for 30 s and stored at –‍20 °C for one hour. The subsequent sample preparation followed the same protocol as the brain samples.

#### Non-targeted metabolomics

The analysis was performed using an HPLC-Q-TOF system, consisting of an Agilent 1290 Infinity HPLC system (RRID:SCR_019375) and an Agilent 6550 iFunnel Q-TOF LC/MS (RRID:SCR_019433). Two different columns with orthogonal separation properties were used: the Waters ACQUITY UPLC BEH C18 column for non-polar and mild-polar metabolites, and the Agilent InfinityLab Poroshell 120 HILIC-Z for polar metabolites. The mobile phases consisted of water or acetonitrile, each mixed with 0.1% formic acid. The column temperature was set at 30 °C with an injection volume of 10 μL using an autosampler. Between each injection, the needle was washed three times with 70% water/30% acetonitrile/0.1% formic acid.

The positive and negative ion modes were generated by an electrospray ionization source. The Q-TOF was calibrated prior to analysis in accordance with the manufacturer’s recommendations. The MS/MS system used the Auto MS/MS mode. For MS1, the mass range was 50–‍1000 m/z with an acquisition rate of two spectra per second. MS2 covered a mass range of 30–‍1000 m/z, with a recording rate of seven spectra per second. The specified collision energies were 10 V, 20 V, and 40 V.

#### Data processing

The obtained data were analyzed using the MassHunter Workstation Qualitative Analysis software (version 12.0, Agilent, RRID:SCR_016657). Metabolites were initially identified using Auto-Select Compound Mining. This method incorporates an extraction algorithm that uses the MS/MS spectra to extract the metabolites and assign them a score that reflects the probability of their existence. Metabolites with a score above 80 were compared with the METLIN database (RRID:SCR_010500). Another score reflected the match between the database spectra and the experimental spectra. The resulting dataset contained the identified molecules along with their measured intensity.

Data from the individual measurements (two columns, each in positive and negative mode) were evaluated using an Excel macro. This macro was designed to consolidate multiple annotations within a measurement, thereby combining the four datasets for each sample. The values within the matrix reflected the measured intensity of a metabolite. The output file was used for statistical analysis.

#### Statistical analysis and visualization

Statistical analyses and the graphic plots were created using the MetaboAnalyst 6.0 web application (RRID:SCR_015539). Metabolites with more than 70% missing values in a group were excluded from the dataset. Missing values were replaced with one-fifth of the lowest detected value. Data were transformed to the base-10 logarithm and scaled using the auto-scaling function.

Volcano plots were created using a *p*-value of ≤ 0.05 and a fold change (FC) limit of 2. The volcano plot data were transferred to GraphPad PRISM v.5.00 for visualization. The images of the principal component analyses (PCA), partial least squares discriminant analyses (PLS-DA), heatmaps, and pathway analyses were used as depicted by MetaboAnalyst. Pathway analyses were conducted using the Human Metabolome Database (HMDB, RRID:SCR_007712)^25^ IDs of each metabolite (excluding exogenous compounds). The used pathway database was *Mus musculus* (house mouse, KEGG).

## Results

### Behavioral observations and seizures during the study

Regarding behavioral observations, repetitive jumping and running (stereotypy) were observed in experimental and home cages during the whole study period. A detailed description of seizures events during this study are depicted in Table 1. Three out of 31 Cav^−/−^ mice exhibited seizures, two in the Cav^−/−^_LF group and one in the Cav^−/−^_HF group. These seizure events lasted for 5‍–15 s. One mouse experienced three seizures, the other two mice experienced one seizure each during week 19. Most seizure events were described by freezing of movement and myoclonus. For one mouse, barrel-rolling was observed. After the seizures, the mice appeared not affected and no adverse events or behavior were reported during the following days.

**Table 1:** Seizure events during the study. Seizure events during the study were recorded in detail. The table depicts the mouse’s group, the time point, the number, the duration, and the description of the seizure events.

Group	Seizure during week	Number of seizures	Duration of seizure [s]	Description of seizure
Cav^−/−^_LF	19	1	5	freezing, myoclonus, immediate recovery
Cav^−/−^_LF	19	3	5, 5, 10	freezing, myoclonus, immediate recovery
Cav^−/−^_HF	19	1	15	freezing, myoclonus, barrel-rolling, immediate recovery

### Cav^−/−^ mice show differences in activity, anxiety, and exploratory behavior

The absence of the α_1B_-subunit in Cav^−/−^ mice was confirmed using western blot and proteomic analyses (Supplementary Fig. 1–2). Weight data can be found in the Supplementary Fig. 3, as detailed weight analyses for the comparison between Cav^−/−^ and WT mice were performed in our previous study^18^ and did not differ from the data obtained here. The same is true for the non-mendelian distribution of the Cav^−/−^ genotype during breeding. This phenomenon was already described in the previous study^18^, therefore it is not discussed here.

**Fig. 1:**
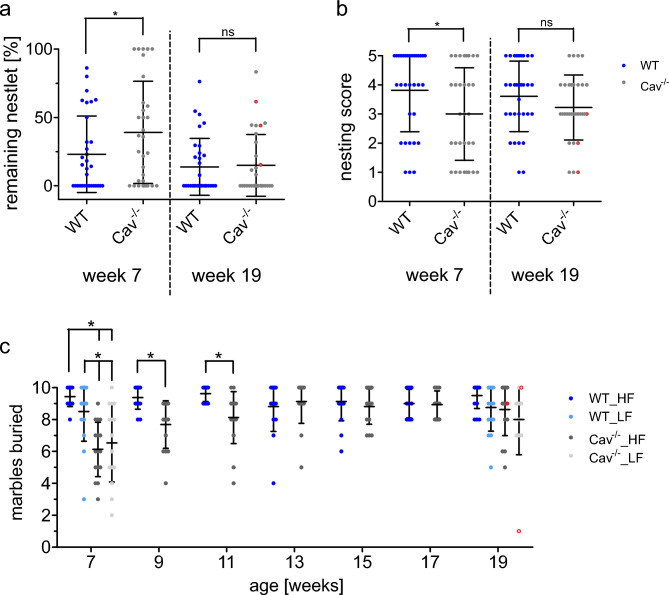
Nesting behavior and marble burying of Cav^−/−^ and WT mice. **a**–**b** At the start (week 7) and end (week 19) of the study a nesting test was performed. The remaining nestlet (% of initial weight) and the nesting score (quality) are presented as mean ± SD. Mice that experienced seizures are marked with a red outline from the time on the seizures appeared. Statistical analyses were performed using two-way ANOVA. (**a**) F_interaction_(1,122) = 2.271, *p* = 0.138; F_group_(1,122) = 2.991, *p* = 0.086; F_age_(1,122) = 11.279, *p* = 0.001. (**b**) F_interaction_(1,122) = 0.797, *p* = 0.374; F_group_(1,122) = 6.199, *p* = 0.014; F_age_(1,122) = 0.002, *p* = 0.962. Post-hoc Holm-Sidak tests identified significant differences between WT and Cav^−/−^ mice (A: *p = 0.024; B: *p = 0.018). **c** Marbles buried at least ¾ were counted. Data are presented as mean ± SD. Statistical analyses were performed using two-way RM ANOVA for weeks 7 to 19 (WT_HF vs. Cav^−/−^_HF), and two-way ANOVA at week 7 and 19 (all groups). Weeks 7–‍19: F_interaction_(6,180) = 8.967, *p* < 0.001; F_group_(1,180) = 27.428, *p* < 0.001; F_age_(6,180) = 4.958, *p* < 0.001. Weeks 7 + ‍19: F_interaction_(3,118) = 3.632, *p* = 0.015; F_group_(3,118) = 12.309, *p* < 0.001; F_age_(1,118) = 12.644, *p* < 0.001. Post-hoc Holm-Sidak tests were performed when differences between the groups were detected (*p < 0.05). (a–‍b) (Cav^−/−^:16♂:15♀, WT: 16♂:16♀). (c) (Cav^−/−^_LF:8♂:7♀, Cav^−/−^_HF: 8♂:8♀, WT_LF: 8♂:8♀, WT_HF: 8♂:8♀). HF = high frequency. LF = low frequency. ns = non-significant.

An examination of the data from the nesting test at the start (week 7) and the end of the study (week 19) revealed no significant differences between the HF and LF groups of WT and Cav^−/−^ mice. Consequently, the data for the genotypes were pooled (Fig. 1a–‍b). The original graphs that depict the four distinct groups are given in the Supplementary Fig. 4. At week 7, 43.75% of the WT mice used the whole nestlet material to build a nest. For Cav^−/−^ mice, this percentage was reduced by half (~ ‍25.81%). The observed difference at week 7 was significant for the percentage of remaining nestlet (*p = 0.024) and the quality score of the built nest (*p = 0.018). At week 19, the proportion of Cav^−/−^ mice that utilized the whole nestlet (~ ‍58.06%) was comparable to that of WT mice (~ ‍59.38%). The percentages of remaining nestlet and the nesting scores exhibited no significant differences between the genotypes.

In the marble burying test (Fig. 1c), the WT_HF and WT_LF groups exhibited a higher number of marbles buried during the initial test week compared to both Cav^−/−^ groups (WT_HF: 9.44 ± 0.61, WT_LF: 8.5 ± 1.80, Cav^−/−^_HF: 6.13 ± 1.65, Cav^−/−^_LF: 6.35 ± 2.50). In weeks 9 and 11, WT_HF mice buried a significantly higher number of marbles than Cav^-/-^_HF mice (9.38 ± 0.70 vs. 7.69 ± 1.45, *p* < 0.001; 9.63 ± 0.48 vs. 8.13 ± 1.58, *p* < 0.001, respectively). In the following weeks, no significant differences were observed between the HF groups. In the final test week, no significant differences were detected between the test frequencies or between the genotypes.

**Fig. 2:**
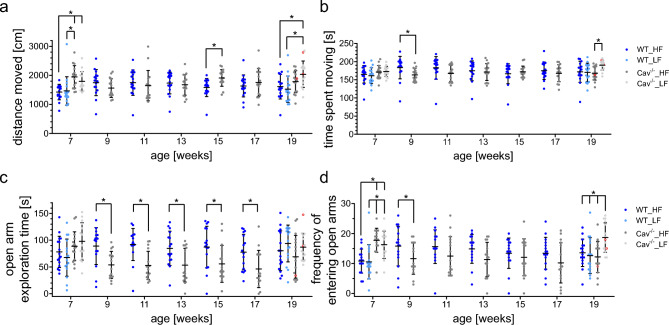
Zero maze of Cav^−/−^ and WT mice. **a–d** Mice were recorded for 5 min in the elevated zero maze. The distance moved, time spent moving, open arm exploration time, and frequency of entering open arms are presented as mean ± SD. Mice that experienced seizures are marked with a red outline from the time on the seizures appeared. Statistical analyses were performed using two-way RM ANOVA for weeks 7 to 19 (WT_HF vs. Cav^−/−^_HF), and two-way ANOVA at week 7 and 19 (all groups). (**a**) Weeks 7–‍19: F_interaction_(6,180) = 5.620, *p* < 0.001; F_group_(1,180) = 1.392, *p* = 0.247; F_age_(6,180) = 0.255, *p* = 0.957. Weeks 7 + 19: F_interaction_(3,118) = 1.615, *p* = 0.190; F_group_(3,118) = 9.523, *p* < 0.001; F_age_(1,118) = 1.311, *p* = 0.255. (**b**) Weeks 7–‍19: F_interaction_(6,180) = 2.861, *p* = 0.011; F_group_(1,180) = 0.651, *p* = 0.426; F_age_(6,180) = 0.776, *p* = 0.590. Weeks 7 + 19: F_interaction_(3,118) = 1.119, *p* = 0.313; F_group_(3,118) = 3.183, *p* = 0.026; F_age_(1,118) = 3.992, *p* = 0.048. (**c**) Weeks 7–‍19: F_interaction_(6,180) = 3.710, *p* = 0.002; F_group_(1,180) = 9.388, *p* = 0.005; F_age_(6,180) = 1.959, *p* = 0.074. Weeks 7 + 19: F_interaction_(3,118) = 2.887, *p* = 0.039; F_group_(3,118) = 1.147, *p* = 0.333; F_age_(1,118) = 0.008, *p* = 0.930. (**d**) Weeks 7–‍19: F_interaction_(6,180) = 6.631, *p* < 0.001; F_group_(1,180) = 1.105, *p* = 0.302; F_age_(6,180) = 1.327, *p* = 0.247. Weeks 7 + 19: F_interaction_(3,118) = 5.225, *p* = 0.002; F_group_(3,118) = 9.346, *p* < 0.001; F_age_(1,118) = 0.221, *p* = 0.640. Post-hoc Holm Sidak tests were performed when differences between the groups were detected (*p < 0.05). (Cav^−/−^_LF: 8♂:7♀, Cav^−/−^_HF: 8♂:8♀, WT_LF: 8♂:8♀, WT_HF: 8♂:8♀). HF = high frequency. LF = low frequency.

For the time spent moving of the zero maze (Fig. 2b), no statistical differences, which persisted over several test weeks, were identified between genotypes or frequencies. For the distance moved (Fig. 2a), Cav^−/−^_HF and Cav^−/−^_LF mice exhibited a significantly increased distance moved compared with WT_HF and WT_LF mice at week 7. From week 9 to 17, the HF groups did not differ (except at week 15). At week 19, only Cav^−/−^_LF mice exhibited a statistically significant increase in distance moved compared with both WT groups. A similar behavior was observed in the frequency of entering the open arms (Fig. 2d): At week 7, Cav^−/−^ mice showed a significantly increased entry frequency to the open arms in comparison with WT mice. At week 19, only Cav^−/−^_LF mice significantly entered the open arms more frequently. Interestingly, WT_HF mice spent more time exploring the open arms (Fig. 2c) than Cav^−/−^_HF mice between weeks 9 and 17. Significant sex differences in the zero maze are displayed in Table 2.

**Table 2:** Sex differences in the zero maze. Sex differences (male vs. female) at different ages and averaged over levels of genotype (WT_HF, WT_LF, Cav^−‍/−^_HF, Cav^−‍/−^_LF) were analyzed using three‑way ANOVA and post‑hoc Holm‑Sidak tests. The significant weeks (*p* < 0.05) during the study period are shown for the open arm exploration of the zero maze. HF = high frequency. LF = low frequency. ns = non‑significant.

**Sex differences**				
	WT_HF	WT_LF	Cav^−/−^_HF	Cav^−‍/−^_LF
open arm exploration time	ns	19	ns	ns

**Fig. 3:**
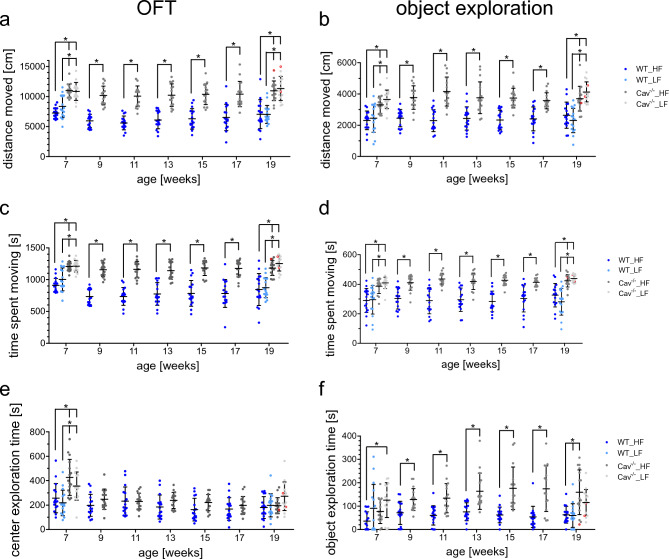
Open field test and object exploration test of Cav^−/−^ and WT mice. **a-f** The mice were recorded for 30 min in the arena without (OFT) and with an object (object exploration) in the center. The distance moved, time spent moving, and center/object exploration are presented as mean ± SD. Mice that experienced seizures are marked with a red outline from the time on the seizures appeared. Statistical analyses were performed using two-way RM ANOVA for weeks 7 to 19 (WT_HF vs. Cav^−/−^_HF), and two-way ANOVA at week 7 and 19 (all groups). (**a**) Weeks 7–‍19: F_interaction_(6,180) = 0.399, *p* = 0.879; F_group_(1,180) = 76.883, *p* < 0.001; F_age_(6,180) = 5.830, *p* < 0.001. Weeks 7 + 19: F_interaction_(3,118) = 1.685, *p* = 0.174; F_group_(3,118) = 49.123, *p* < 0.001; F_age_(1,118) = 0.824, *p* = 0.366. (**b**) Weeks 7–‍19: F_interaction_(6,180) = 2.299, *p* = 0.037; F_group_(1,180) = 47.460, *p* < 0.001; F_age_(6,180) = 2.103, *p* = 0.055. Weeks 7 + 19: F_interaction_(3,118) = 1.182, *p* = 0.320; F_group_(3,118) = 33.692, *p* < 0.001; F_age_(1,118) = 4.159, *p* = 0.044. (**c**) Weeks 7–‍19: F_interaction_(6,180) = 1.465, *p* = 0.193; F_group_(1,180) = 87.203, *p* < 0.001; F_age_(6,180) = 4.477, *p* < 0.001. Weeks 7 + 19: F_interaction_(3,118) = 1.856, *p* = 0.141; F_group_(3,118) = 46.043, *p* < 0.001; F_age_(1,118) = 2.980, *p* = 0.087. (**d**) Weeks 7–‍19: F_interaction_(6,180) = 1.458, *p* = 0.195; F_group_(1,180) = 50.949, *p* < 0.001; F_age_(6,180) = 1.1508, *p* = 0.178. Weeks 7 + 19: F_interaction_(3,118) = 1.011, *p* = 0.390; F_group_(3,118) = 31.040, *p* < 0.001; F_age_(1,118) = 3.309, *p* = 0.071. (**e**) weeks 7–‍19: F_interaction_(6,180) = 3.617, *p* = 0.002; F_group_(1,180) = 7.971, *p* = 0.008; F_age_(6,180) = 14.019, *p* < 0.001. Weeks 7 + 19: F_interaction_(3,118) = 5.463, *p* = 0.001; F_group_(3,118) = 8.728, *p* < 0.001; F_age_(1,118) = 28.207, *p* < 0.001. (**f**) Weeks 7–‍19: F_interaction_(6,180) = 2.475, *p* = 0.025; F_group_(1,180) = 38.557, *p* < 0.001; F_age_(6,180) = 5.751, *p* < 0.001. Weeks 7 + 19: F_interaction_(3,118) = 4.263, *p* = 0.007; F_group_(3,118) = 8.300, *p* < 0.001; F_age_(1,118) = 1.976, *p* = 0.162. Post-hoc Holm-Sidak tests were performed when differences between the groups were detected (*p < 0.05). (Cav^−/−^_LF: 8♂:7♀, Cav^−/−^_HF: 8♂:8♀, WT_LF: 8♂:8♀, WT_HF: 8♂:8♀). HF = high frequency. LF = low frequency.

As illustrated in Fig. 3a–‍d, Cav^−/−^ mice exhibited an increased distance moved in comparison with WT mice, irrespective of test frequency or the placement of objects into the arena. The exploration time of the center during the OFT (Fig. 3e) showed that Cav^-/-^ mice explored the center significantly more only at week 7. Subsequently, no differences were observed between WT_HF and Cav^−/−^_HF mice. At week 19, the Cav^−/−^_LF group exhibited the longest center exploration time (non-significant). The placement of an object in the arena (Fig. 3f) significantly increased the exploration time of the center for Cav^−/−^ mice compared with WT mice across all test weeks. This is in contrast to the center exploration time observed during the OFT. Significant sex and part differences of the OFT and object exploration test are displayed in Table 3.

**Table 3:** Holm-Sidak tests for the total body weight and weight change. Sex (male vs. female) and part differences (part 1 vs. part 2) at different ages were analyzed using two-way ANOVA and post-hoc Holm-Sidak tests for each genotype (WT_HF, WT_LF, Cav^−‍/−^_HF, Cav^−‍/−^_LF). The significant weeks (*p* < 0.05) during the study period are shown for the time spent moving and object exploration time of the object exploration test. HF = high frequency. LF = low frequency. ns = non-significant.

**Sex differences**				
	WT_HF	WT_LF	Cav^−/−^_HF	Cav^−‍/−^_LF
time spent moving, object exploration test	11, 13, 15	ns	ns	ns

Part differences				
	WT_HF	WT_LF	Cav^−/−^_HF	Cav^−‍/−^_LF
object exploration time	ns	ns	19	ns

### Cav^−/−^ mice exhibited increased inflammation in the hippocampus and cortex

Given the absence of significant differences between the HF and LF groups in both genotypes in the behavioral tests (except for certain habituation effects observed in the final test week), subsequent analyses were conducted exclusively between WT and Cav^−/−^ mice in whole (for histology: n(WT) = 11; n(Cav^−/−^) = 13).

**Fig. 4:**
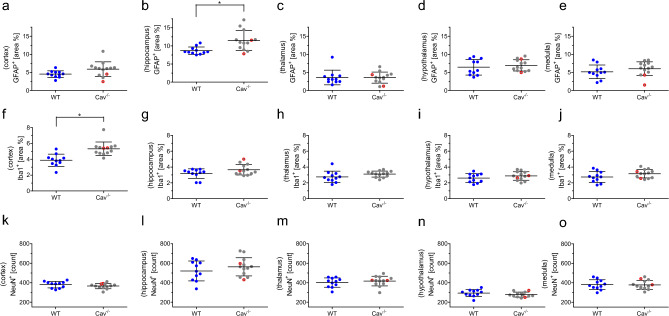
Quantification of astrocytes, activated microglia, and neurons in the brain of Cav^−/−^ and WT mice. **a-o** Astrocytes were quantified using GFAP in the cortex, hippocampus, thalamus, hypothalamus, and medulla (area of the vestibular nucleus). Mice that experienced seizures are marked with a red outline from the time on the seizures appeared. Cav^−/−^ mice were compared with WT mice. Data are presented as mean ± SD and analyzed using t-tests and rank sum tests. (**a**) *p* = 0.077, (**b**) *p = 0.003, (**c**) *p* = 0.791, (**d**) *p* = 0.451, (**e**) *p* = 0.281, (**f**) *p < 0.001, (**g**) *p* = 0.079, (**h**) *p* = 0.142, (**i**) *p* = 0.239, (**j**) *p* = 0.112, (**k**) *p* = 0.267, (**l**) *p* = 0.310, (**m**) *p* = 0.471, (**n**) *p* = 0.226, (**o**) *p* = 0.931. (Cav^−/−^: 7♂:6♀, WT: 5♂:6♀).

The histological analyses of the brain were conducted in the cortex, hippocampus, thalamus, hypothalamus, and medulla (area of the vestibular nucleus). Brain regions were selected according to Bauer and colleagues^26^. The majority of brain regions exhibited no difference in the percentage of the GFAP-positive area of astrocytes (Fig. 4a–‍e, Supplementary Fig. 5–9). However, the hippocampus of Cav^−/−^ mice exhibited an increased fraction of GFAP-positive astrocytes compared with WT mice (WT: 8.68% ± 1.02%, Cav^−/−^: 11.50% ± 2.77%, *p = 0.003).

A significant difference was observed in the area of activated microglia of Cav^−/−^ mice in comparison with WT mice in the cortex (Fig. 4f, Supplementary Fig. 10–14). The Iba1-positive area exhibited a significant increase with 5.33% ± 0.86% in Cav^−/−^ mice compared with 3.88% ± 0.77% in WT mice (*p* < 0.001). The remaining brain regions demonstrated no significant differences between the genotypes (Fig. 4g–‍j).

To examine potential neurodegeneration, the number of NeuN-positive neurons was quantified in the brain regions (Fig. 4k–‍o, Supplementary Fig. 10–14). No significant differences were detected between WT and Cav^−/−^ mice in any of the investigated brain regions regarding the NeuN count.

### Analysis of brain metabolites in Cav^−/−^ mice

**Fig. 5:**
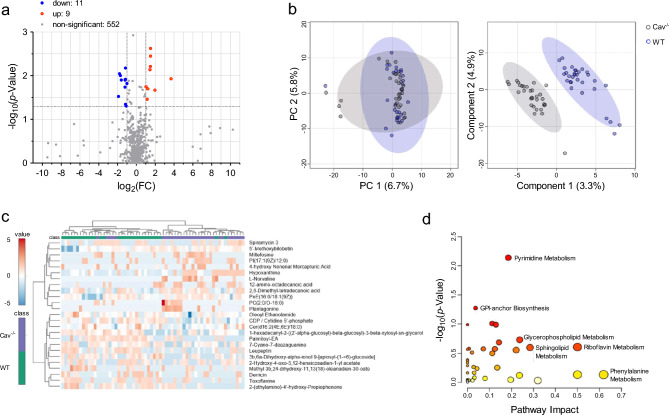
Analysis of differential metabolites in the brain of Cav^−/−^ mice. Untargeted metabolomics of Cav^**−/−**^ and WT brains were performed. (**a**) In the volcano plot, blue indicates downregulated metabolites (*p* < 0.05; FC < 0.5), and red indicates upregulated metabolites (*p* < 0.05; FC > 2) in Cav^−/−^ mice. Grey dots represent metabolites without significant differences between the genotypes. (**b**) A PCA score plot and PLS-DA score plot were created. Circles represent the 95% confidence intervals. (**c**) The heatmap shows the top 25 metabolites by VIP score which allow class discrimination. (**d**) The significantly altered pathways are displayed as circles. Darker colors represent lower *p*-values, the circle size represents the impact score. The most impacted pathways were annotated. **a-d** All analyses were performed using MetaboAnalyst 6.0. FC = fold change. PC = principal component.

Figure 5a presents a volcano plot illustrating the differentially regulated brain metabolites for the Cav^−/−^/WT comparison. A total of 572 metabolites were detected, of which 11 were significantly downregulated (*p* < 0.05, FC < 0.5) and 9 were significantly upregulated (*p* < 0.05, FC > 2) in Cav^−/−^ mice. An overview of these differentially regulated metabolites can be found in Table 4.

**Table 4:** Differential metabolites between Cav^−/−^ and WT mice in the brain. In comparison to WT mice, 9 compounds were upregulated and 11 compounds were downregulated in the brains of Cav^−/−^ mice. FC = fold change.

Metabolite	FC	*p*-Value
N4-Acetylsulfapyridine	0.267	0.030
Leupeptin	0.287	0.009
Palmitoyl-EA	0.307	0.010
Methyl 3b,24-dihydroxy-11,13(18)-oleanadien-30-oate	0.328	0.013
Derricin	0.430	0.018
Cer(d16:2(4E,6E)/18:0)	0.433	0.013
2-(ethylamino)−4'-hydroxy-Propiophenone	0.441	0.012
Dapsone N-sulfamate	0.446	0.045
Toxoflavine	0.446	0.007
PE-Cer(d16:1(4E)/21:0)	0.473	0.050
3b,6a-Dihydroxy-alpha-ionol 9-[apiosyl-(1- > 6)-glucoside]	0.496	0.015
Miltefosine	2.025	0.018
N(alpha)-t-Butoxycarbonyl-L-leucine	2.202	0.035
Spiramycin 3	2.328	0.020
Hypoxanthine	2.715	0.007
L-Norvaline	2.811	0.006
Plantagonine	2.823	0.002
PI(17:1(9Z)/12:0)	2.841	0.004
4-hydroxy Nonenal Mercapturic Acid	3.916	0.022
PC(2:0/O-16:0)	12.891	0.012

A PCA revealed that both genotypes exhibited a predominantly similar metabolic profile (Fig. 5b). However, one Cav^**−/−**^ mouse and four WT mice did not cluster with the rest of their respective genotype. The PLS-DA (Fig. 5b) confirmed that these mice did not fit or only barely fitted into the 95% confidence interval. The analysis also distinctly differentiated between Cav^−/−^ and WT mice using brain metabolomics.

VIP scores (Supplementary Table 1) were used to generate a heatmap of the top 25 discriminating metabolites in the brain (Fig. 5c). No metabolites were excluded; only those deemed relevant are discussed. The heatmap, similar to the PCA, demonstrated that the samples were not distinctly clustered into the two genotypes. The endogenous compounds cytidine 5’-phosphate, palmitoylethanolamide (palmitoyl-EA, PEA), the phosphatidylinositol PI(17:1(9Z)/12:0), oleoyl ethanolamide (OEA), and 2,6-dimethyl-tetradecanoic acid were identified as the five metabolites with the highest VIP scores. The group of glycerophospholipids and other lipids were represented with more than one metabolite.

The glycerophospholipid biosynthesis was observed to be differentially regulated between Cav^**−/−**^ and WT brains in the pathway analysis (Fig. 5d). The most significantly altered pathway was the pyrimidine metabolism, followed by glycosylphosphatidylinositol (GPI)-anchor biosynthesis, sphingolipid metabolism, and riboflavin metabolism. Impact scores and *p*-values for the annotated pathways are given in the Supplementary Table 2.

### Analysis of plasma metabolites in Cav^−/−^ mice

As previously mentioned, Cav^−/−^ mice could develop seizures, as observed during the present study and our previous study^18^. Given the higher seizure incidence documented in the previous study compared with the present study, we aimed to investigate this discrepancy in seizure events using a comparative analysis of the LC–MS/MS data. However, the previous study focused on plasma data (i.e., no comparison of brain data possible). Given that during the previous study seizures only occurred in the second part of the study, the 9 Cav^−/−^ mice participating in this second part are included in the analysis here.

**Fig. 6:**
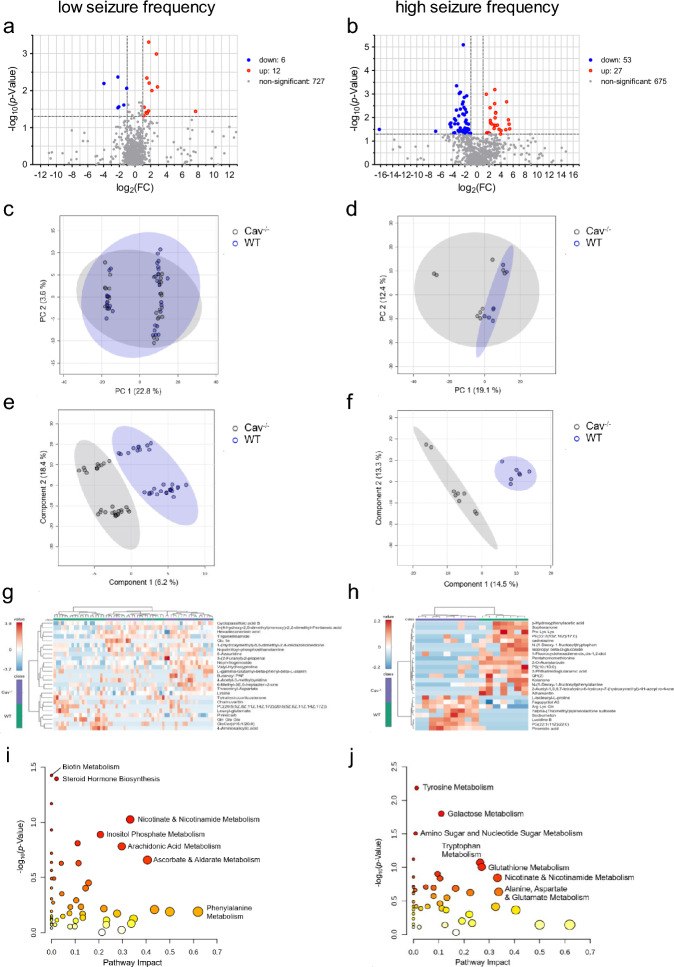
Analysis of differential metabolites in the plasma of Cav^−/−^ mice. Untargeted metabolomics of Cav^**−/−**^ and WT plasma, collected during this study and a previous study^18^, were performed. This comparison was conducted because during the previous study Cav^−/−^ mice had more seizure events than during this study. (**a-b**) In the volcano plots, blue indicates downregulated metabolites (*p* < 0.05; FC < 0.5), and red indicates upregulated metabolites (*p* < 0.05; FC > 2) in Cav^−/−^ mice. Grey dots represent metabolites without significant differences between the genotypes. (**c-d**) PCA score plots and (**e-f**) PLS-DA score plots were created. Circles represent the 95% confidence intervals. (**g-h**) The heatmaps show the top 25 metabolites by VIP score which allow class discrimination. (**i-j**) The significantly altered pathways are displayed as circles. Darker colors represent lower *p*-values, the circle size represents the impact score. The most impacted pathways are annotated. (a–‍j) All analyses were performed using MetaboAnalyst 6.0. FC = fold change. PC = principal component.

Figure 6a–‍b compares the differentially regulated plasma metabolites of the present study (low seizure frequencies) and the previous study (high seizure frequencies). In the present study, 18 differentially regulated metabolites (*p* < 0.05; 0.5 > FC > 2) were detected when comparing Cav^−/−^ and WT mice. In the previous study, 80 differentially regulated compounds were identified, exhibiting a greater number of significantly altered metabolites. These metabolites are listed in Table 5.

**Table 5:** Differential plasma metabolites between Cav^−/−^ and WT mice. Compared with WT mice, 6 metabolites were downregulated and 12 metabolites were upregulated in the plasma of Cav^−/−^ mice of this study. For a previous study^18^, 53 metabolites were downregulated and 27 metabolites were upregulated in the plasma of Cav^−/−^ mice compared with WT mice. The current study exhibited low seizure frequencies of Cav^−/−^ mice, and the previous study exhibited high seizure frequencies of Cav^−/−^ mice. FC = fold change.

Current study		
Metabolite	FC	***p***-value
Tetrahydrocorticosterone	0.065	0.006
Gln Glu Glu	0.221	0.029
4-Aminosalicylic acid	0.223	0.004
Leucyl-glutamate	0.243	0.027
Chamuvaritin	0.374	0.025
PC(20:5(5Z,8Z,11Z,14Z,17Z)/20:5(5Z,8Z,11Z,14Z,17Z))	0.485	0.009
Dihydrocaffeic acid 3-O-glucuronide	2.181	0.048
1-(Hydroxymethyl)−5,5-dimethyl-2,4-imidazolidinedione	2.247	0.028
5-Azauridine	2.686	0.039
Threoninyl-Aspartate	2.845	0.005
Oleamide	2.944	0.041
Hexadecanedioic acid	3.321	0.036
Glu Ile	3.349	0.001
Valyl-Hydroxyproline	3.483	0.006
N-palmitoyl-phosphoethanolamine	4.414	0.010
4-Acetyl-3-methylpyridine	6.645	0.001
Butanoyl PAF	7.256	0.008
3-(2-Furanyl)−2-propenal	206.750	0.036

Previous study		
Metabolite	FC	***p***-value
L-Sorbose	0.00001	0.032
2-Protocatechoylphloroglucinolcarboxylate	0.009	0.039
6-Hydroxyshogaol	0.045	0.019
Nummularine F	0.050	0.017
PE-Cer(d14:2(4E,6E)/20:1(11Z)(2OH))	0.051	0.025
Oleamide	0.070	0.045
Pro Lys Lys	0.076	0.011
Gemeprost	0.077	0.044
N-(1-Deoxy-1-fructosyl)isoleucine	0.079	0.019
3-Methylbutyl 2-furanbutanoate	0.087	0.013
N-(1-Deoxy-1-fructosyl)tryptophan	0.095	0.005
Sophoranone	0.096	0.001
9-Tricosene	0.113	0.018
Tyr Thr Lys	0.116	0.036
N-(1-Deoxy-1-fructosyl)phenylalanine	0.125	0.008
3-(4-Isopropylphenyl)propanal	0.127	0.040
2-Acetyl-1,5,6,7-tetrahydro-6-hydroxy-7-(hydroxymethyl)−4H-azepine-4-one	0.131	0.001
4S,5R-antillatoxin A	0.137	0.038
Trigonellinamide	0.142	0.037
(3beta,8beta)−3-Hydroxy-7(11)-eremophilen-12,8-olide	0.149	0.028
Isopropyl beta-D-glucoside	0.150	0.001
p-Hydroxyphenylacetic acid	0.151	0.004
N-D-Glucosylarylamine	0.153	0.037
PC(O-2:0/O-1:0)	0.178	0.037
Panaxynol linoleate	0.191	0.038
cadralazine	0.191	0.003
Athamantin	0.199	0.007
Anhalonidine	0.199	0.041
PS(10:0/10:0)	0.206	0.002
2-O-Acetylarbutin	0.208	< 0.001
V-PYRRO/NO	0.213	0.034
PE(22:2(13Z,16Z)/17:0)	0.225	0.004
PE(22:1(11Z)/15:0)	0.229	0.037
2-Phthalimidoglutaramic acid	0.236	0.001
QH(2)	0.238	0.008
PE-Cer(d16:2(4E,6E)/21:0)	0.251	0.046
9alpha-Fluoro-11beta,16alpha,17alpha,21-tetrahydroxypregn-4-ene-3,20-dione	0.253	0.028
1-Fluorocyclohexadiene-cis,cis-1,2-diol	0.264	0.005
Phosphoric acid	0.267	0.015
N-Nitrososarcosine	0.288	0.013
Kolanone	0.302	0.006
Todralazine	0.304	0.018
PE(22:6(4Z,7Z,10Z,13Z,16Z,19Z)/19:1(9Z))	0.306	0.038
3,7-Dimethyl-2E,6E-decadien-1,10-dioic acid	0.307	0.041
Byakangelicin	0.312	0.032
Pentahomomethionine	0.313	0.001
PE(22:6(4Z,7Z,10Z,13Z,16Z,19Z)/20:4(8Z,11Z,14Z,17Z))	0.326	0.030
10-Acetoxyligustroside	0.340	0.030
3beta-3-Lupanol	0.344	0.046
N-Acetyl-a-neuraminic acid	0.375	0.031
2-Fluorocyclohexadiene-cis,cis-1,2-diol-1-carboxylate	0.387	0.019
Ile Lys Arg	0.392	0.033
Hypaphorine	0.482	0.047
Arg Lys Gln	2.961	0.001
Stigmatellin Y	3.136	0.045
Thymine	3.578	0.045
N-stearoyl valine	3.939	0.045
Fludrocortisone	4.242	0.049
Alprenolol	4.407	0.047
Lucidine B	4.559	0.004
PE(19:1(9Z)/0:0)	4.664	0.013
Gln Asn Ser	4.820	0.017
Coumarin	5.517	0.019
3-Fluorocyclohexadiene-cis,cis-1,2-diol-1-carboxylate	7.302	0.029
Phenylalanyl-Aspartate	7.646	0.021
Fagopyritol A3	7.703	0.003
PG(22:1(11Z)/22:0)	7.841	0.001
Piromidic acid	8.150	0.010
7α-(Thiomethyl)spironolactone sulfoxide	8.596	0.006
L-isoleucyl-L-proline	8.734	0.007
C75	8.794	0.022
Embelin	10.710	0.021
1-Oleoyl-2-acetyl-sn-glycerol	12.851	0.030
Glc-GP(18:0/20:4(5Z,8Z,11Z,14Z))	15.431	0.050
(E)−2-Methyl-2-buten-1-ol O-beta-D-Glucopyranoside	16.120	0.036
4-Amino-2-hydroxylamino-6-nitrotoluene	28.031	0.033
Secbumeton	30.811	0.002
ADP-D-ribose	37.605	0.013
Secobarbital	40.693	0.019
N,N-diisopropyl-3-nitrobenzamide	43.602	0.030

Notably, the PCA and the PLS-DA of the current study’s plasma metabolites (Fig. 6c, e) revealed a perfect separation into part 1 and part 2 of the study (see “2.1.2 Study Design”) regardless of the genotype or HF/LF group. Separation of the genotypes was only possible in the PLS-DA, and separation of part 1 and 2 remained within the genotype. In the previous study with high seizure frequencies (Fig. 6 d, f.)^18^, the metabolites of Cav^−/−^ mice exhibited a greater scattering of individual samples in comparison with WT mice. However, the genotypes demonstrated an overlap in the PCA, and clear differentiation was only possible in the PLS-DA.

The previous study with high seizure frequencies^18^ was the only one to achieve classification into the Cav^−/−^ and WT genotypes (Fig. 6h). The top 25 metabolites by VIP score clustered in upregulated metabolites or downregulated metabolites, depending on the genotype. The top differentiating metabolites were PG(22:1(11Z)/22:0), isopropyl beta-D-glucoside, arginyl-lysyl-glutamine (Arg Lys Gln), PE(22:2(13Z,16Z)/17:0), and p-Hydroxyphenylacetic acid. The present study identified endogenous metabolites that differentiated between Cav^−/−^ and WT mice (Fig. 6g), including glutamyl-isoleucine (Glu Ile), tetrahydrocorticosterone, threoninyl-aspartate, N-palmitoyl phosphoethanolamine, and valyl-hydroxyproline. The VIP scores for all top 25 compounds from both studies can be found in the Supplementary Table 3. The metabolic profile of Cav^−/−^ mice with an active seizure phenotype from the previous study^18^ was distinct from that of WT mice. The metabolic profile of Cav^−/−^ mice that were predisposed for seizures, as in the present study, was not clearly separated from WT mice, showing intra-genotype differences compared with the previous study.

The pathway analysis of the current study (Fig. 6i) exhibited a significantly different regulated steroid hormone biosynthesis between Cav^−/−^ and WT mice, with a minimal pathway impact. Pathways with a higher impact that did not reach significance included the nicotinate and nicotinamide metabolism, arachidonic acid metabolism, and ascorbate and aldarate metabolism. In the previous study with higher seizure frequencies (Fig. 6j), the tyrosine and galactose metabolisms were significantly altered between Cav^−/−^ and WT mice. A number of pathways exhibited a higher impact score; however, they did not reach statistical significance: tryptophan metabolism, glutathione metabolism, nicotinate and nicotinamide metabolism, and alanine, aspartate and glutamate metabolism. The nicotinate and nicotinamide metabolism was significantly altered in Cav^−/−^ across both studies. The impact scores and *p*-values for the annotated pathways are given in the Supplementary Table 4.

## Discussion

In this study, the Ca_V_2.2 KO mouse line, as described by Saegusa and colleagues^13^, was investigated by exploring characteristics that had not yet been observed due to a single testing rather than a longitudinal study design. We investigated whether a higher frequency of experimental stress would be sufficient to induce epilepsy-like seizures similar to those previously observed^18^. The discussion includes the most relevant behavioral results, excluding the nesting test and marble burying test. Additionally, given that this study is longitudinal, only the statistically significant changes persistent over several experimental weeks are going to be discussed. Significant differences that were only observed for a single time point were considered a natural variability between and inside the study groups.

The KO of the α_1B_-subunit was confirmed by western blot and proteomic analysis (Supplementary Fig. 1–2), suggesting a complete Ca_V_2.2 deficiency in Cav^−/−^ mice. Furthermore, the behavioral differences observed between Cav^−/−^ and WT mice were not attributed to differences in the proteome.

Saegusa et al. demonstrated that Cav^−/−^ mice entered the open arms more frequently and spent more time in the open arms than WT mice in the plus maze. They concluded that Cav^−/−^ mice exhibited reduced anxiety-related behaviors in environments associated with heights and/or open spaces^13^. We used an elevated zero maze (Fig. 2) to eliminate the center zone of the plus maze that cannot be assigned to either the open or the closed arms. This set up confirmed Saegusa’s observations. However, upon repetition of the test, WT mice explored and entered the open arms more frequently than Cav^−/−^ mice. This genotype difference in longitudinal tests suggested a habituation effect, because Cav^−/−^_LF mice entered the open arms more frequently than any other group in the last test week.

Saegusa and colleagues interpreted the increased time spent moving and total distance moved in the OFT as reduced anxiety-related behavior^13^. The present study confirmed the hyperactive behavior of Cav^−/−^ mice throughout all test weeks, regardless of test frequency (Fig. 3a, 3c). The center exploration time was increased only at week 7 (Fig. 3e). The increased mobility exhibited by Cav^−/−^ mice might not exclusively be attributed to a reduced anxiety-related behavior, but rather to hyperactivity. Moreover, some mice showed signs of hyperactivity and/or stereotypy in their cages, which corresponds to our previous study^18^. Another Ca_V_2.2 KO mouse line exhibited an increased spontaneous locomotor activity in the OFT^15^ and an increased locomotor activity in the activity wheel test^16^. These observations suggest that hyperactivity is a pivotal characteristic of Ca_V_2.2-deficient mice.

The results of the object exploration test (Fig. 3f) showed an increased exploratory behavior in Cav^−/−^ mice, as opposed to a solely reduced anxiety-related behavior. In general, the processes of object exploration and recognition are regulated by the hippocampus^27^. The increased object exploration exhibited by Cav^−/−^ mice indicates alterations in neuronal circuits of this specific brain region. Ino and colleagues performed a Y-maze with a Ca_V_2.2 KO line, thereby demonstrating that Cav^−/−^ mice exhibited an intact short-term memory^12^. However, the results of the passive avoidance test suggested a dysregulated long-term memory^16^. Based on these observations, the increased object exploration of Cav^−/−^ mice may be attributed, at least in part, to an impaired long-term memory.

In the present study, three Cav^−/−^ mice exhibited single seizure events (brief, immediate recovery). These were also observed during our previous study, in which five out of nine Cav^−/−^ mice exhibited repetitive seizures. Out of these five mice, three were euthanized due to a poor health status following a seizure. Possibly, the severity of seizures was caused by an increased test frequency in part 2 relative to part 1^18^. The observations suggested that conducting multiple behavioral and motoric tests per day on a weekly basis resulted in experimental stress, thereby inducing an increased seizure frequency in Cav^−/−^ mice. Therefore, the hypothesis of the present study suggested that Cav^−/−^_HF mice would exhibit a greater number of seizure events compared with Cav^−/−^_LF mice. However, for the present study, a reduced number of motoric tests per day and a reduced test frequency were chosen to ensure animal welfare. This modification may have resulted in a decline of seizure events compared with the previous study. Consequently, subsequent analyses were performed with pooled Cav^−/−^ and WT groups, investigating a possible pre-seizure phenotype of Cav^−/−^ mice.

Inflammation and neurodegeneration are hallmarks of epilepsy^28–30^. GFAP-positive astrocytes were increased in the hippocampus of Cav^−/−^ mice (Fig. 4b), a brain region involved in the transfer of short-term memory and spatial information into long-term memory^31^. This increased astrocyte signal suggested an altered hippocampal function, leading to an impaired long-term memory and increased exploration, as evidenced by the object exploration test (Fig. 3). Furthermore, activated microglia were increased in the prefrontal cortex of Cav^−/−^ mice (Fig. 4f). This brain region is involved in decision-making upon learned categorization or impulses^32–34^, as well as in social interactions and the formation of social memory^35,36^. The presence of microgliosis in this brain region indicated alterations of these networks, as evidenced by the hyperactivity and excessive object exploration of Cav^−/−^ mice, and by the voluntary termination of the pole and wire hanging test in our previous study^18^. Neurodegeneration in Cav^−/−^ mice was not observed in the investigated brain regions (Fig. 4k–‍o), possibly because neuronal loss occurs later in epilepsy pathology, whereas hippocampal gliosis is characterized as an early symptom^37^. Given that only three mice in the present study were observed to have seizure events, neurodegeneration may not yet manifest in the entire Cav^−/−^ group.

The presynaptic location of Ca_V_2.2^2^ suggests that a KO alters the excitability of neurons and, consequently, their metabolic profile. The differential regulation of metabolites in Cav^−/−^ mice (Fig. 5a, Table 4) included the significantly downregulated lipid PEA (Fig. 5c), which has been previously shown to have anti-inflammatory^38^ and anti-nociceptive effects in mice^39^. In an epileptic mouse model, brain levels of PEA were downregulated, and PEA supplementation before epileptogenesis reduced seizure frequency and severity^40^. The observed downregulation of PEA in Cav^−/−^ mice may indicate an increased susceptibility to develop seizures. PE-Cer(d16:1(4E)/21:0) was also downregulated in Cav^−/−^ mice (Table 4). This sphingolipid ceramide was identified as a member of myocardial specific lipids^41^. Elevated heart rates and mean arterial blood pressure were measured in other Ca_V_2.2-deficient mouse lines, suggesting a dysregulated sympathetic regulation^12,14^. In general, ceramides are components of complex sphingolipids. Depletion of ceramide synthase 2 resulted in progressive myoclonic epilepsy, accompanied by altered sphingolipid levels^42^. This study connects the observed decrease in ceramides and an epilepsy syndrome.

4-hydroxy nonenal mercapturic acid (HNE-MA) was upregulated in the brain of Cav^−/−^ mice (Table 4). This metabolite has been used as a biomarker for oxidative stress in lipid peroxidation^43,44^. The heatmap (Fig. 5c) demonstrated that several differentially regulated glycerophospholipids and lipids were detected in Cav^−/−^ mice, which calls for lipidomics in the Ca_V_2.2 KO mouse line. Furthermore, hypoxanthine was also upregulated in Cav^−/−^ mice (Table 4), and it is discussed as a potential biomarker in ischemic stroke because it could induce vascular injury and disruption of the blood–brain-barrier^45^. Hypoxanthine levels were also consistently upregulated in various models of pharmacologically induced seizures^46^. These metabolites provide compelling evidence of the seizure-prone phenotype of Cav^−/−^ mice.

Despite the inability to differentiate between the Cav^−/−^ and WT genotype by brain metabolites (Fig. 5b–‍c), the most effective differentiator by VIP-score was the nucleotide cytidine 5’-phosphate. It is part of the pyrimidine salvage pathway^47^ that is represented in the Cav^−/−^/WT comparison in the pathway analysis, the pyrimidine biosynthesis (Fig. 5d). This finding may indicate an altered nucleotide processing, which could explain the non-mendelian distribution of the Cav^−/−^ genotype in our studies (present and previous study^18^) and the described mortality by Saegusa and colleagues^13^. The second significantly altered pathway in the brain of Cav^−/−^ mice is the GPI-anchor biosynthesis (Fig. 5d), which is located in the ER and represents post-translational modifications^48^. Glutamatergic neurons of Cav^−/−^ mice may exhibit dysregulated calcium concentrations, which in turn could influence the GPI-anchor biosynthesis via the Ca^2+^-sensitive chaperone calnexin^49^.

The repetitive seizures observed in our previous study^18^ may have triggered an overall higher detection of differentially regulated metabolites in the plasma of Cav^−/−^ mice compared with the present study (Fig. 6a–‍b). The most relevant endogenous metabolites associated with a low frequency of seizures were butanoyl platelet-activating factor (PAF), oleamide, and tetrahydrocorticosterone (Table 5, Fig. 6g). First, butanoyl PAF was upregulated in the plasma of patients with ischemic strokes^50^, and demonstrated to be a damage-associated phospholipid, capable of activating the NLRP3 inflammasome^51^. The upregulation of PAF in Cav^−/−^ mice suggests a potential link between the Ca_V_2.2 KO and the occurrence of seizures and inflammation. Second, oleamide administration has been demonstrated to reduce novelty-induced behaviors and to achieve an antidepressant-like effect^52^. Furthermore, oleamide administration in mice with pentylenetetrazole-induced seizures demonstrated a protective effect^53^. The upregulation observed in Cav^−/−^ mice suggests the presence of an endogenous mechanism that mitigates stereotypy and seizures. Third, tetrahydrocorticosterones have been demonstrated to bind to glucocorticoid receptors as anti-inflammatory agents similar to corticosterone^54,55^. Its downregulation in the plasma of Cav^−/−^ mice suggests a suppression of the corticosterone pathway in relation to inflammation or, more broadly, to a dysregulated steroid hormone pathway. This dysregulation was reflected in the significantly altered steroid hormone biosynthesis in Cav^−/−^ mice of the present study (Fig. 6i), suggesting an inflammatory regulation, as this pathway has been identified as significantly altered in other inflammatory diseases^56,57^. The most significantly altered pathway, although with a low impact score, was the biotin metabolism (Fig. 6i). The key metabolite L-lysine, or more precisely the L-lysine degradation, has been associated with epilepsy disorders^58,59^. However, given the significantly different regulation of other amino acids and their combinations in Cav^−/−^ mice (Fig. 6g), these mice may exhibit a generalized amino acid dysregulation.

In the plasma of Cav^−/−^ mice with high seizure frequencies^18^, p-hydroxyphenylacetic acid was downregulated (Fig. 6h, Table 5). The metabolite has previously been detected in the urine of an infant with developmental retardation, seizures, and spasticity^60^. The polyphenol functions as a modulator of monoamine oxidase, an enzyme that has been targeted in the treatment of depression and neurodegenerative diseases^61,62^. The amino acid thymine was upregulated in Cav^−/−^ mice. This metabolite has been detected in patients diagnosed with mild cognitive impairment and Alzheimer’s disease, and it has been correlated with two score systems evaluating cognitive decline^63^. This finding suggests a cognitive deficit in Cav^−/−^ mice with seizures, a hypothesis that has been previously postulated for this mouse line^16^. N-stearoyl valine was upregulated in the plasma of Cav^−/−^ mice with seizures. Conversely, this plasma metabolite was downregulated in a chronic neuropathic pain mouse model^64^. A reduced reaction to neuropathic pain is a hallmark of the Cav^−/−^ mouse line^13^, which suggests that the upregulation of N-stearoyl valine reflects a protective mechanism against neuropathic pain. Detected metabolites were predominantly lipids and amino acids (Table 5). The most differentially regulated pathway of the previous study^18^ was the tyrosine metabolism (Fig. 6j). This pathway suggests a neuromodulating effect Cav^−/−^ mice, given that elevated levels of tyrosine have been associated with inflammation, mitochondrial dysfunction, and neurotransmitter dysfunction^65^. The latter directly connects the tyrosine metabolism and the Ca_V_2.2 channel. The galactose metabolism, and amino sugar and nucleotide sugar metabolism were found to be significantly altered in Cav^−/−^ mice with seizures (Fig. 6j). Both pathways are associated with epilepsy: First, the galactose pathway in the plasma of patients with mesial temporal lobe epilepsy^66^; second, the amino sugar and nucleotide sugar metabolism in the cerebrospinal fluid of patients with different epilepsy phenotypes^59^. While the metabolites of the previous study^18^ effectively distinguished the genotypes, the present study exhibited no such differentiation (Fig. 6g–‍h). This observation may reflect the timeline of epileptogenesis^29,37^, in which the present study represents the early phase with a predominant focus on inflammation, while the previous study represents the phase of active seizure events. As a disease, syndrome, or disorder progresses, the more the metabolome may undergo significant changes compared with an unaffected individual.

In addition to several significantly altered metabolites, we identified a few that are of exogenous or environmental origin, such as actinomycetes. Their biological relevance remains to be explored further. Furthermore, despite the implementation of rigorous quality control measures to minimize batch effects, such as randomized run order and pooled QC samples, metabolomics data can still be susceptible to technical variations. Lastly, functional validation and longitudinal follow-up were beyond the scope of this study, but are essential next steps in determining the significance of the observed metabolic changes. Despite these limitations, our findings provide important groundwork for future targeted investigations in this area.

Overall, the Ca_V_2.2 channel is imperative for the correct signal transmission between neurons, and a Ca_V_2.2 deficiency can result in behavioral and metabolic changes in mice. Seizures were also observed, especially when Cav^−/−^ mice participated in weekly behavioral and motoric tests^18^. This finding is consistent with other presynaptic components that have been identified as epilepsy-related proteins, including Ca_V_2.1^67^. We could not determine a cause for the development of seizures in the Cav^−/−^ genotype, but we hypothesize that experimental stress can lead to a seizure-prone phenotype. Electrophysiological recordings, analyses of brain metabolites, and histochemistry at the exact time point of the seizure event would further characterize the link between the Ca_V_2.2 KO and the development of seizures. Nevertheless, affected mice could be characterized by histological and metabolomic changes that are consistent with the literature about epilepsy-like seizures.

## Conclusion

This characterization study of the Ca_V_2.2 KO mouse line by Saegusa and colleagues^13^ was conducted to elucidate behavioral differences to WT mice, which were observed in a previous study^18^, but not yet examined in detail. We could confirm the Cav^−/−^ mice to be hyperactive and less anxious in novel conditions. The repetition of tests changed the reduced anxiety-linked behavior, but not the increased mobility. An increased level of exploration was observed throughout the whole study period. Histology revealed an increased inflammation in the cortex and the hippocampus. The metabolomic analyses suggested the Ca_V_2.2 KO mouse model to be prone for dysregulation of the presynapse and potentially to seizures. Consequently, when using this mouse line in pain-related studies, it is imperative to consider the susceptibility of Cav^−/−^ mice to experience seizures.

## Supplementary Information

Supplementary Information.

## Data Availability

The LC–MS/MS metabolomics data have been deposited to the MetaboLights^68^ repository with the study identifier MTBLS14319 (https://www.ebi.ac.uk/metabolights/MTBLS14319). All other datasets generated and analyzed during the current study are available from the corresponding author on reasonable request.

## Abbreviations

Ca^2+^: Calcium ions
Ca_V_2.2: Voltage-gated calcium channel 2.2
Cav^−/−^: Mice with Ca_V_2.2 knockout
DTT: Dithiothreitol
FC: Fold change
GFAP: Glial fibrillary acidic protein
GPI: Glycosylphosphatidylinositol
HF: High frequency
Iba1: Ionized calcium-binding adapter molecule 1
KO: Knockout
LC–MS/MS: Liquid chromatography-tandem mass spectrometry
LF: Low frequency
NeuN: Neuronal nuclei
OEA: Oleoyl ethanolamide
OFT: Open field test
PAF: Platelet-activating factor
PCA: Principal component analysis
PEA: Palmitoylethanolamide
PFA: Paraformaldehyde
PLS-DA: Partial least squares discriminant analysis
RM: Repeated measures
ROI: Region of interest
VGCC: Voltage-gated calcium channel
WT: Wild-type
