# Supplementary material for: In-depth characterization of the CaV2.2-knockout mouse line
Source: Sci Rep. 2026 Jul 9;16:21418. doi: 10.1038/s41598-026-60827-w (PMC13351052; doi:10.1038/s41598-026-60827-w)
Supplement: Supplementary file 1 — Supplementary Information. [file 41598_2026_60827_MOESM1_ESM.docx]

# Supplementary information

| Article | In-depth characterization of the Ca_V_2.2-knockout mouse line with epilepsy‑like seizures |
| --- | --- |
| Journal | Scientific Reports |
| Authors | Katharina Wintz, Felix Schumacher, Clemens Pfeiffer, Maja Schäfer, Ian Gering, Sarah Schemmert, Antje Willuweit, Janine Kutzsche |
| Corresponding Author | Janine Kutzsche  Institut für Physikalische Biologie, Heinrich Heine University Düsseldorf, Faculty of Mathematics and Natural Sciences, Universitätsstraße 1, 40225 Düsseldorf, Germany  e-mail: j.kutzsche@fz-juelich.de |


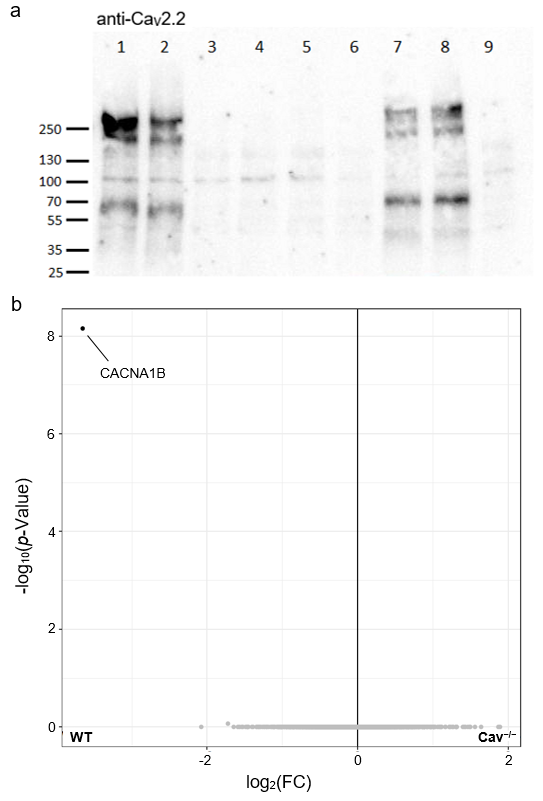
**Supplementary Fig. 1**

Absence of the α_1B_‑subunit in Cav^−/−^ mice. **a** A Western blot analysis revealed the absence of the Ca_V_2.2 channel (~‍240 kDa) in Cav^−/−^ (samples 3–‍6, 9) but not WT (samples 1, 2, 7, 8) brain homogenates. The blot was cropped to size and labeled with numbers; otherwise, the original blot image remained unchanged. The original images are depicted in Supplementary Fig. 2. **b** Proteomics demonstrated that the only significantly altered protein in the Cav^−/−^/WT comparison is the CACNA1B protein, the α_1B_‑subunit of the Ca_V_2.2 channel. A protein is considered significantly altered at *p* < 0.05 and FC < 0. FC = fold change


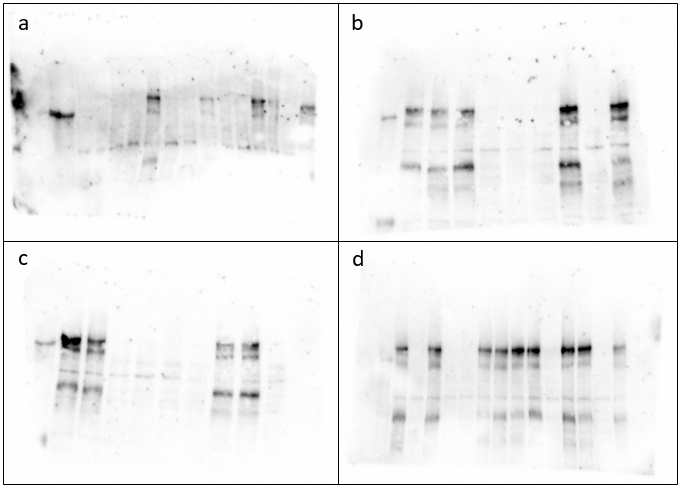
**Supplementary Fig. 2**

**a–‍d** Original western blots of brain homogenates for the detection of the α_1B_‑subunit. Brain homogenates of the mice were analyzed by western blot with an antibody against the α_1B_‑subunit (ACC‑002, Alomone Labs, Israel). Identification of Cav^−/−^ and WT genotypes was possible by comparing bands around 240 kDa, which is the molecular weight of the Ca_V_2.2 channel.

**Supplementary Fig. 3**

Comparison of body weight progression in Cav^−/−^ and WT mice. The mice were weighed 3‑times per week. **a–‍b** The total weight and the percentage of the initial weight are presented as mean ± SD. Statistical analyses were performed using RM two­‑way ANOVA. **a** F_interaction_(39,748) = 1.044, *p* = 0.399; F_group_(3,748) = 1.628, *p* = 0.193; F_age_(13,748) = 682.697, *p* < 0.001. **b** F_interaction_(39,767) = 4.120, *p* < 0.001; F_group_(3,767) = 1.583, *p* = 0.203; F_age_(13,767) = 875.611, *p* < 0.001. Post‑hoc Holm‑Sidak tests revealed significant differences (*p* < 0.05) in weight change between WT_HF and Cav^−/−^_LF mice (week 20), and WT_LF vs. Cav^−‍/−^_LF mice (weeks 13–14). (Cav^−/−^_LF 8♂:7♀, Cav^−/−^_HF 8♂:8♀, WT_LF 8♂:8♀, WT_HF 8♂:8♀). HF = high frequency. LF = low frequency.

**Supplementary Fig. 4**

Nesting behavior of Cav^−/−^_HF, Cav^−/−^_LF, WT_HF, and WT_LF mice. At the start (week 7) and the end (week 19) of the study a nesting test was performed. **a–‍d** The remaining nestlet (% of initial weight) and the nesting score (quality) are presented as mean ± SD. Mice that experienced seizures are marked with a red outline from the time on the seizures appeared. There were no significant differences (ns) between the genotypes (Cav^−/−^ vs. WT) or the test frequencies (HF vs. LF). Statistical analyses were performed using one‑way ANOVA. **a** *p* = 0.321; **b** *p* = 0.206; **c** *p* = 0.503; **d** *p* = 0.275. As no significant differences were detected, post‑hoc tests were not performed. (Cav^−/−^_LF 8♂:7♀, Cav^−/−^_HF 8♂:8♀, WT_LF 8♂:8♀, WT_HF 8♂:8♀). HF = high frequency. LF = low frequency.


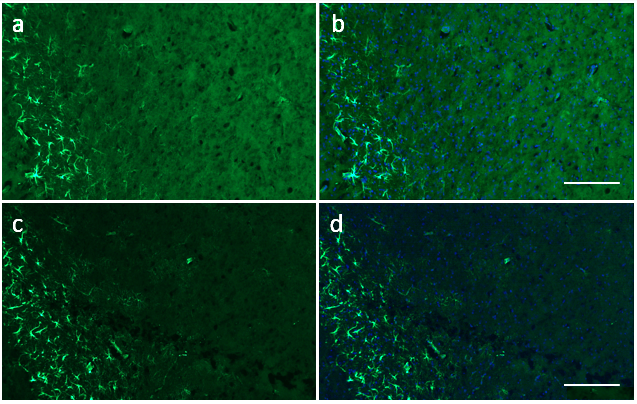
**Supplementary Fig. 5**

Histology of GFAP in the brain, cortex. The signal of GFAP (green, activated astrocytes) in the cortex were compared between **a–‍b** WT and **c–‍d**Cav^−/−^ mice. **b, d** Overlays additionally show DAPI (blue). Scale: 200 μM.


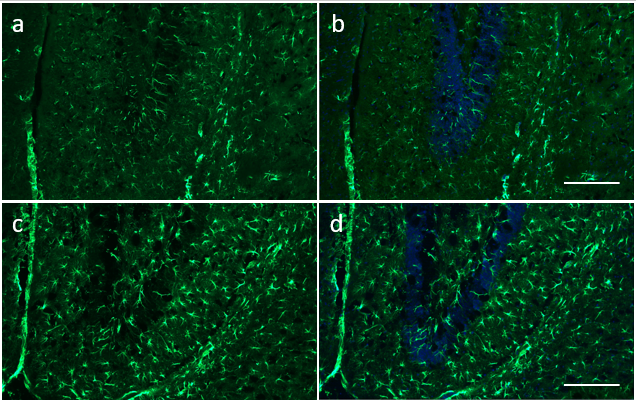
**Supplementary Fig. 6**

Histology of GFAP in the Brain, Hippocampus. The signal of GFAP (green, activated astrocytes) in the hippocampus were compared between **a–‍b** WT and **c–‍d** Cav^−/−^ mice. **b, d** Overlays additionally show DAPI (blue). Scale: 200 μM.


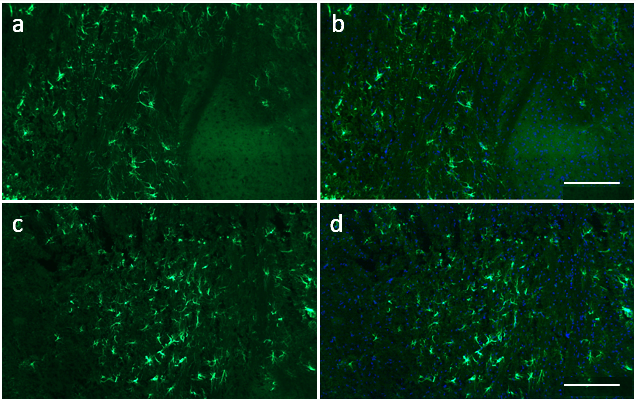
**Supplementary Fig. 7**

His**t**ology of GFAP in the brain, hypothalamus: The signal of GFAP (green, activated astrocytes) in the hypothalamus were compared between **a–‍b** WT and **c–‍d** Cav^−/−^ mice. **b, d** Overlays additionally show DAPI (blue). Scale: 200 μM.


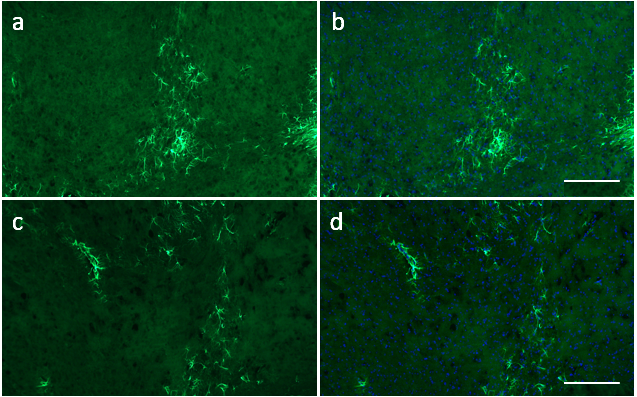
**Supplementary Fig. 8**

Histology of GFAP in the brain, thalamus: The signal of GFAP (green, activated astrocytes) in the thalamus were compared between **a–‍b** WT and **c–‍d** Cav^−/−^ mice. **b, d** Overlays additionally show DAPI (blue). Scale: 200 μM.


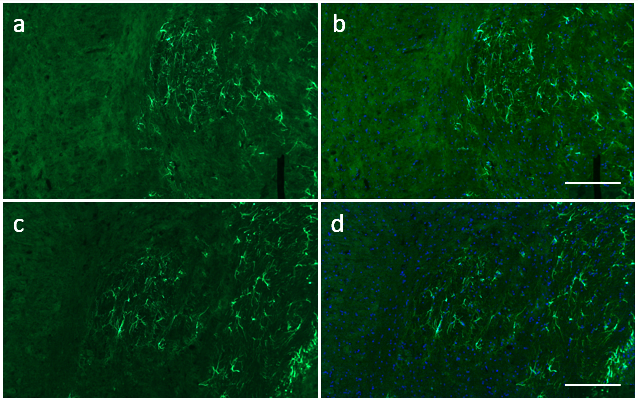
**Supplementary Fig. 9**

Histology of GFAP in the brain, medulla**.** The signal of GFAP (green, activated astrocytes) in the medulla were compared between **a–‍b** WT and **c–‍d** Cav^−/−^ mice. **b, d** Overlays additionally show DAPI (blue). Scale: 200 μM.


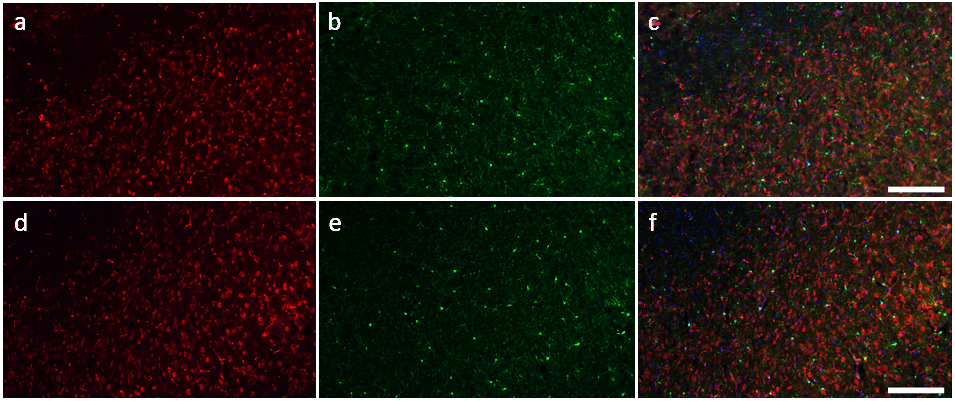
**Supplementary Fig. 10**

Histology of NeuN and Iba1 in the brain, cortex. The signals of NeuN (red, neuronal nuclei) and Iba1 (green, activated microglia) in the cortex were compared between **a–‍c** WT and **d–‍f** Cav^−/−^ mice. **c, f** Overlays additionally show DAPI (blue). Scale: 200 μM.


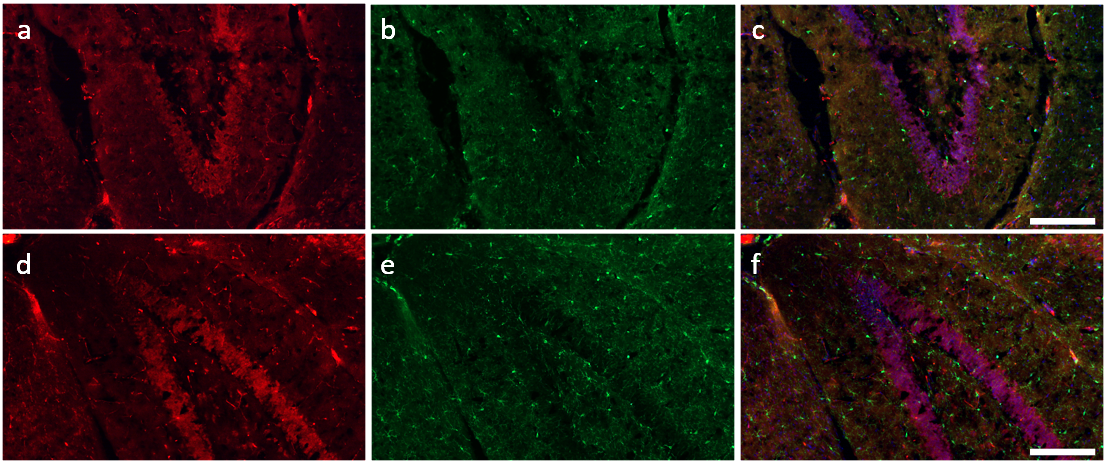
**Supplementary Fig. 11**

Histology of NeuN and Iba1 in the brain, hippocampus. The signals of NeuN (red, neuronal nuclei) and Iba1 (green, activated microglia) in the hippocampus were compared between **a–‍c** WT and **d–‍f** Cav^−/−^ mice. **c, f** Overlays additionally show DAPI (blue). Scale: 200 μM.


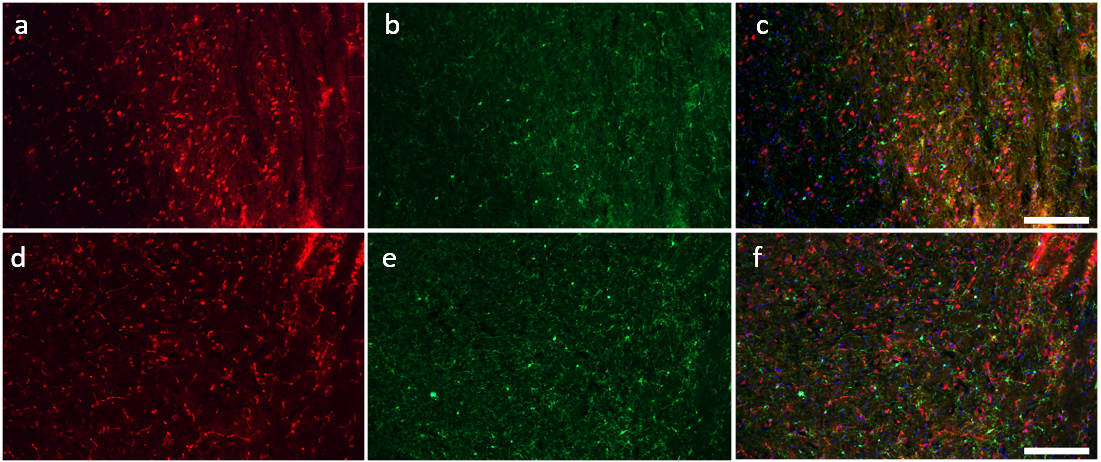
**Supplementary Fig. 12**

Histology of NeuN and Iba1 in the brain, hypothalamus. The signals of NeuN (red, neuronal nuclei) and Iba1 (green, activated microglia) in the hypothalamus were compared between **a–‍c** WT and **d–‍f** Cav^−/−^ mice. **c, f** Overlays additionally show DAPI (blue). Scale: 200 μM.


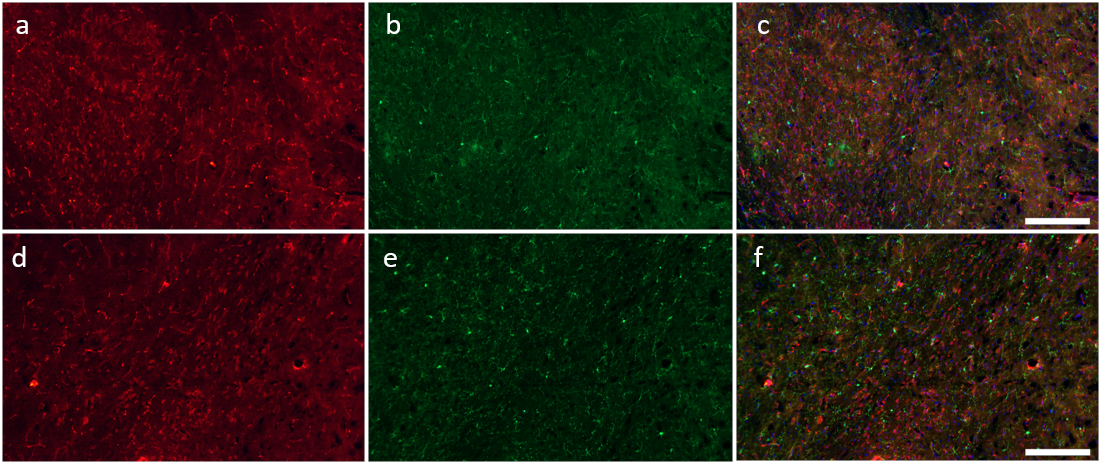
**Supplementary Fig. 13**

Histology of NeuN and Iba1 in the brain, thalamus. The signals of NeuN (red, neuronal nuclei) and Iba1 (green, activated microglia) in the thalamus were compared between **a–‍c** WT and **d–‍f** Cav^−/−^ mice. **c, f** Overlays additionally show DAPI (blue). Scale: 200 μM.


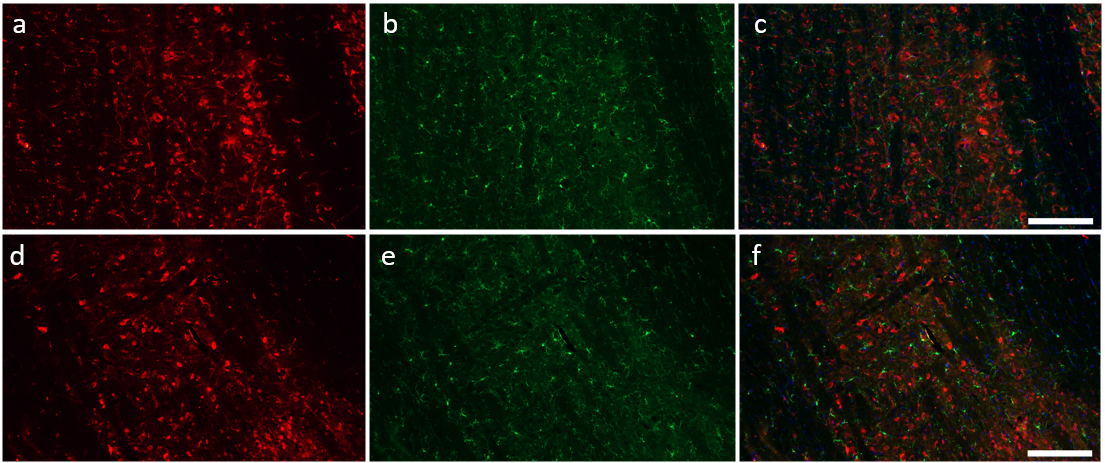
**Supplementary Fig. 14**

Histology of NeuN and Iba1 in the brain, medulla**.** The signals of NeuN (red, neuronal nuclei) and Iba1 (green, activated microglia) in the medulla were compared between **a–‍c** WT and **d–‍f** Cav^−/−^ mice. **c, f** Overlays additionally show DAPI (blue). Scale: 200 μM.

**Supplementary Table 1 Top 25 differentially regulated metabolites between Cav^−/−^ and WT mice in the brain**

The table depicts the top 25 metabolites in the brain by VIP score to discriminate between the Cav^−/−^ and WT genotype.

| **Metabolite** | **VIP Score** |
| --- | --- |
| CDP / Cytidine 5'-diphosphate | 3.000 |
| Plantagonine | 2.911 |
| L-Norvaline | 2.861 |
| Palmitoyl-EA | 2.755 |
| PI(17:1(9Z)/12:0) | 2.606 |
| Oleoyl Ethanolamide | 2.564 |
| 2,6-Dimethyl-tetradecanoic acid | 2.495 |
| Cer(d16:2(4E,6E)/18:0) | 2.483 |
| PC(2:0/O-16:0)[U] | 2.469 |
| 2-Hydroxy-4-oxo-5,12-heneicosadien-1-yl acetate | 2.461 |
| Leupeptin | 2.452 |
| 3b,6a-Dihydroxy-alpha-ionol 9-[apiosyl-(1->6)-glucoside] | 2.418 |
| PA(P-16:0/20:5(5Z,8Z,11Z,14Z,17Z)) | 2.381 |
| PE(19:1(9Z)/0:0) | 2.347 |
| 2-(ethylamino)-4'-hydroxy-Propiophenone | 2.342 |
| Hypoxanthine | 2.288 |
| Miltefosine | 2.229 |
| Polidocanol | 2.226 |
| Toxoflavine | 2.185 |
| 7-Cyano-7-deazaguanine | 2.172 |
| 5'-Methoxybilobetin | 2.168 |
| 1-hexadecanyl-2-((2'-alpha-glucosyl)-beta-glucosyl)-3-beta-xylosyl-sn-glycerol | 2.167 |
| PG(16:0/18:1(9Z)) | 2.164 |
| PnE(16:0/18:1(9Z)) | 2.124 |
| O-Phosphorylethanolamine | 2.117 |

**Supplementary Table 2 Pathway analysis of Cav^−/−^ brain metabolites**

The table depicts annotated, altered pathways in the Cav^−/−^ brain compared with WT brain.

| **Pathway** | **Match Status** | ***p*‑Value** | **Impact Factor** |
| --- | --- | --- | --- |
| Pyrimidine metabolism | 5/39 | 0.007 | 0.186 |
| Glycosylphosphatidylinositol (GPI)-anchor biosynthesis | 2/32 | 0.053 | 0.037 |
| Glycerophospholipid metabolism | 4/36 | 0.186 | 0.237 |
| Sphingolipid metabolism | 4/32 | 0.253 | 0.285 |
| Riboflavin metabolism | 2/4 | 0.248 | 0.500 |
| Phenylalanine metabolism | 2/10 | 0.737 | 0.619 |

**Supplementary Table 3 Top 25 differentially regulated metabolites between Cav^−/−^ and WT mice in the plasma**

The table depicts the top 25 metabolites in the plasma by VIP score to discriminate between the Cav^−/−^ and WT genotype. The plasma samples were collected during the current study and a previous study [1]. The current study exhibited low seizure frequencies of Cav^−/−^ mice, and the previous study exhibited high seizure frequencies of Cav^−/−^ mice.

| **Current Study** | |
| --- | --- |
| **Metabolite** | **VIP Score** |
| Glu Ile | 3.490 |
| 4-Acetyl-3-methylpyridine | 3.211 |
| Tetrahydrocorticosterone | 3.027 |
| Threoninyl-Aspartate | 2.944 |
| N-palmitoyl-phosphoethanolamine | 2.858 |
| Valyl-Hydroxyproline | 2.857 |
| 4-Aminosalicylic acid | 2.851 |
| Butanoyl PAF | 2.729 |
| Leucyl-glutamate | 2.608 |
| PC(20:5(5Z,8Z,11Z,14Z,17Z)/20:5(5Z,8Z,11Z,14Z,17Z)) | 2.508 |
| nephritogenoside | 2.405 |
| Chamuvaritin | 2.369 |
| L-gamma-Glutamyl-beta-phenyl-beta-L-alanine | 2.363 |
| 3-(2-Furanyl)-2-propenal | 2.334 |
| Gln Glu Glu | 2.321 |
| Isobutyryl carnitine | 2.304 |
| 5-(4-hydroxy-2,5-dimethylphenoxy)-2,2-dimethyl-Pentanoic acid | 2.300 |
| Traumatic Acid | 2.295 |
| Trigonellinamide | 2.276 |
| Cyclopassifloic acid B | 2.240 |
| GlcCer(d15:1/20:0) | 2.206 |
| Oleamide | 2.202 |
| 6-Methyl-3E,5-heptadien-2-one | 2.197 |
| 1,8-Diazabicyclo[5.4.0]undec-7-ene | 2.177 |
| Dihydrocaffeic acid 3-O-glucuronide | 2.165 |
| **Previous Study** | |
| **Metabolite** | **VIP Score** |
| 2-O-Acetylarbutin | 2.701 |
| PG(22:1(11Z)/22:0) | 2.349 |
| Sophoranone | 2.342 |
| 2-Acetyl-1,5,6,7-tetrahydro-6-hydroxy-7-(hydroxymethyl)-4H-azepine-4-one | 2.297 |
| Isopropyl beta-D-glucoside | 2.292 |
| Secbumeton | 2.258 |
| Arg Lys Gln | 2.226 |
| Pentahomomethionine | 2.216 |
| 2-Phthalimidoglutaramic acid | 2.158 |
| PS(10:0/10:0) | 2.142 |
| Fagopyritol A3 | 2.116 |
| Lucidine B | 2.105 |
| cadralazine | 2.083 |
| PE(22:2(13Z,16Z)/17:0) | 2.081 |
| 7alpha±-(Thiomethyl)spironolactone sulfoxide | 2.074 |
| 1-Fluorocyclohexadiene-cis,cis-1,2-diol | 2.062 |
| Athamantin | 2.029 |
| p-Hydroxyphenylacetic acid | 2.028 |
| N-(1-Deoxy-1-fructosyl)tryptophan | 2.011 |
| L-isoleucyl-L-proline | 1.978 |
| QH(2) | 1.956 |
| Kolanone | 1.924 |
| N-Nitrososarcosine | 1.916 |
| N-(1-Deoxy-1-fructosyl)phenylalanine | 1.910 |
| PE(19:1(9Z)/0:0) | 1.901 |

**Supplementary Table 4 Pathway analysis of Cav^−/−^ plasma metabolites**

The table depicts annotated, altered pathways in the Cav^−/−^ plasma compared with WT plasma that was collected during the current study and a previous study [1]. The current study exhibited low seizure frequencies of Cav^−/−^ mice, and the previous study exhibited high seizure frequencies of Cav^−/−^ mice.

| **Current Study** | | | |
| --- | --- | --- | --- |
| **Pathway** | **Match Status** | ***p*‑Value** | **Impact Factor** |
| Biotin metabolism | 1/10 | 0.038 | 0.000 |
| Steroid hormone metabolism | 3/79 | 0.040 | 0.021 |
| Nicotinate and nicotinamide metabolism | 13/15 | 0.094 | 0.333 |
| Inositol phosphate metabolism | 3/30 | 0.129 | 0.207 |
| Arachidonic acid metabolism | 2/43 | 0.165 | 0.297 |
| Ascorbate and aldarate metabolism | 3/10 | 0.220 | 0.405 |
| Phenylalanine metabolism | 2/10 | 0.646 | 0.619 |
| **Previous Study** | | | |
| **Pathway** | **Match Status** | ***p*‑Value** | **Impact Factor** |
| Tyrosine metabolism | 2/42 | 0.007 | 0.013 |
| Galactose metabolism | 2/27 | 0.016 | 0.110 |
| Amino sugar and nucleotide sugar metabolism | 1/42 | 0.031 | 0.008 |
| Tryptophan metabolism | 4/41 | 0.085 | 0.265 |
| Glutathione metabolism | 4/28 | 0.098 | 0.270 |
| Nicotinate and nicotinamide metabolism | 3/15 | 0.143 | 0.332 |
| Alanine, aspartate and glutamate metabolism | 4/28 | 0.232 | 0.337 |

**References**

1. Wintz K, Lechtape PL, Klenzendorf J, Schemmert S, Dingley AJ, Willuweit A, et al. (2026) Elucidation of the influence of the Ca_V_2.2 calcium channel on ALS disease progression in the SOD1*G93A mouse model*.* Neurobiology of Disease. 224:107396.

DOI: 10.1016/j.nbd.2026.107396.
